# From Genes to Pathways: The Molecular Landscape of Systemic Lupus Erythematosus

**DOI:** 10.3390/ijms27104552

**Published:** 2026-05-19

**Authors:** Romana Rashid, Zaida G. Ramirez-Ortiz

**Affiliations:** Department of Medicine, Division of Infectious Diseases and Immunology, University of Massachusetts Chan Medical School, 364 Plantation St. LRB319, Worcester, MA 01605, USA

**Keywords:** systemic lupus erythematosus (SLE), epigenetics, genetics, scavenger receptors, immune response, pathophysiology

## Abstract

Systemic lupus erythematosus (SLE) is a prototypic systemic autoimmune disorder arising from the convergence of genetic susceptibility, epigenetic remodeling, environmental exposures, and dysregulated immune networks. Although traditionally characterized by autoantibody production and immune complex mediated tissue injury, advances in genomics, systems immunology, and multi-omics profiling have revealed that lupus represents a multilayered failure of immune homeostasis driven by interconnected molecular circuits. Genetic variants enriched in regulatory immune enhancers establish a permissive transcriptional landscape that sensitizes innate nucleic acid sensing pathways and interferon signaling. Epigenetic remodeling further amplifies inflammatory transcriptional programs, while environmental triggers such as ultraviolet radiation and viral infection initiate bursts of nucleic acid release and immune activation. Defective apoptotic cell clearance, mediated in part by scavenger receptor dysfunction and complement abnormalities, increases the availability of immunogenic nucleic acids that engage pattern recognition receptors and drive chronic type I interferon production. This interferon-dominated environment rewires immune cell metabolism, alters differentiation trajectories of T and B lymphocytes, and sustains autoreactive immune circuits. Emerging multi-omics studies reveal distinct molecular endotypes defined by interferon signatures, metabolic states, and immune cell composition, highlighting the heterogeneity of disease mechanisms across patients. In this review, we integrate genetic, epigenetic, metabolic, and immunological insights to propose a systems-level model of lupus pathogenesis in which defective debris clearance, nucleic acid sensing, interferon amplification, and metabolic reprograming form a self-reinforcing pathogenic network. Understanding this integrated molecular architecture provides a foundation for biomarker-guided therapeutic strategies and precision medicine approaches aimed at disrupting the key nodes that sustain chronic autoimmunity in SLE.

## 1. Introduction

Systemic lupus erythematosus (SLE) is a chronic systemic autoimmune disorder characterized by the production of autoantibodies directed against nuclear and cytoplasmic antigens and by immune-mediated injury affecting multiple organ systems, including the kidneys, skin, joints, cardiovascular system, and central nervous system, to name a few. SLE exhibits striking clinical heterogeneity, ranging from mild mucocutaneous involvement to severe renal, neurological, and hematological complications. This heterogeneity reflects complex molecular and cellular processes that disrupt immune tolerance and promote chronic inflammation [1,2].

**Figure 1:**
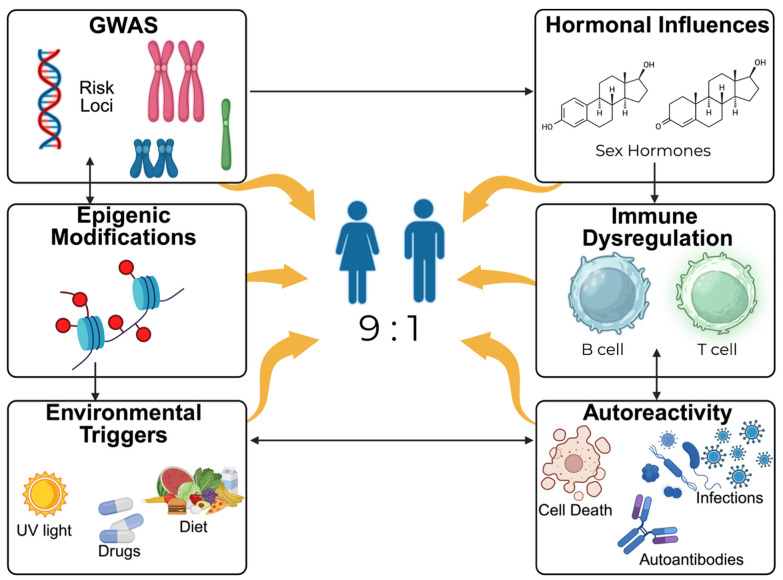
Mechanisms contributing to autoimmune disease development. This schematic illustrates the multifactorial processes underlying autoimmune disease. Genetic risk loci identified by genome-wide association studies (GWAS), epigenetic modifications, and environmental triggers such as UV light, drugs, and diet interact with hormonal influences, including sex hormones, to modulate immune regulation. Immune dysregulation, involving B cells and T cells, promotes autoreactivity characterized by cell death, infections, and the generation of autoantibodies. The black arrows depict whether the processes are unidirectional or bidirectional. Yellow arrows indicate interconnected pathways among these factors, with central depiction of female and male figures highlighting the relative prevalence ratio (9:1) commonly observed in many autoimmune diseases. https://BioRender.com/uwmld0d (accessed on 15 April 2026).

Historically, lupus pathogenesis has been interpreted primarily through the paradigm of autoantibody production and immune complex deposition, in which circulating immune complexes composed of autoantibodies and nucleic acid containing antigens accumulate in tissues and activate complement and Fc receptor signaling pathways (Figure 1). Experimental and clinical studies confirm that immune complexes contribute directly to tissue injury, particularly in lupus nephritis [3]. However, advances in genomics, transcriptomics, and systems immunology have demonstrated that this model represents only one component of a broader network of immune dysregulation. Genome-wide association studies (GWAS) have identified more than one hundred susceptibility loci linked to SLE, most of which reside in noncoding regulatory regions that influence gene expression in immune cells rather than altering protein structure [4,5]. These observations suggest that inherited risk largely operates through altered transcriptional control of immune signaling pathways.

Epigenomic profiling further reveals that lupus-associated variants overlap with enhancer elements responsive to inflammatory transcription factors such as IRF and STAT family members [6,7]. Functional studies indicate that these variants influence chromatin accessibility and transcription factor binding in immune cell populations, including B cells, T cells, and monocytes. Concurrently, defects in apoptotic cell (AC) clearance and nucleic acid degradation pathways permit the accumulation of endogenous DNA and RNA species capable of activating innate immune receptors. Experimental evidence from murine models lacking nucleases such as TREX1 or DNase II demonstrates that failure to degrade self-nucleic acids leads to chronic activation of type I interferon pathways and lupus-like disease phenotypes [8].

These molecular abnormalities converge to produce a pathogenic immune environment characterized by persistent activation of plasmacytoid dendritic cells (pDCs), sustained type I interferon (IFN) production, and expansion of autoreactive B and T lymphocytes. The resulting cytokine milieu promotes differentiation of pathogenic immune cell subsets and supports the generation of high-affinity autoantibodies. Importantly, high dimensional approaches such as single-cell RNA sequencing and spatial transcriptomics now demonstrate that immune dysregulation in lupus is distributed across multiple cellular compartments rather than confined to a single lineage [9]. Distinct transcriptional states have been identified in interferon-responsive monocytes, age-associated B cells, and T peripheral helper cells within patient tissues, suggesting that lupus comprises multiple molecular endotypes rather than a single uniform disease entity [10].

This review synthesizes current knowledge to present a systems-level model of lupus pathogenesis in which genetic risk, epigenetic regulation, nucleic acid sensing, metabolic reprograming, and immune cell interactions form an interconnected network driving chronic autoimmunity. Understanding how these regulatory layers interact is essential for explaining disease heterogeneity and for identifying molecular pathways that may be therapeutically targeted.

## 2. Genetic Architecture of Systemic Lupus Erythematosus

SLE arises from a complex genetic architecture in which numerous common variants of modest effect interact with rare high-impact mutations to shape immune function and disease susceptibility. Evidence for heritable risk has long been supported by familial aggregation studies and twin analyses. Concordance rates among monozygotic twins are substantially higher than among dizygotic twins, yet remain incomplete, indicating that genetic predisposition establishes a permissive background rather than acting as a deterministic cause of disease [11]. Population studies further demonstrate that individuals of African, Asian, and Hispanic ancestry exhibit higher prevalence and often more severe disease manifestations, reflecting differences in allele frequencies and genetic architecture across populations [12].

GWAS have now identified more than 100 loci associated with SLE susceptibility [13]. Remarkably, most of these loci reside in noncoding genomic regions enriched for regulatory elements active in immune cells rather than in protein-coding sequences [4]. Because most SLE-associated variants reside in noncoding regions, their functional effects are primarily mediated through disruption of cis-regulatory elements that control gene transcription [14]. Enhancers and promoters contain specific transcription factor binding motifs, and single-nucleotide variants within these regions can alter binding affinity, thereby modifying recruitment of transcriptional machinery and changing gene expression levels in a cell-type-specific manner [15]. Integrative analyses combining GWAS with epigenomic maps have shown that autoimmune disease risk variants are significantly enriched in active enhancer elements in immune cells, particularly those marked by H3K27ac and DNase I hypersensitivity, indicating localization within accessible regulatory chromatin [16,17]. In SLE, GWAS risk variants are preferentially enriched in enhancer regions active in immune cell subsets, particularly B cells, T cells, and monocytes [4]. Consistent with a direct regulatory role, many SLE-associated variants colocalize with expression quantitative trait loci, demonstrating that these polymorphisms quantitatively influence gene expression in relevant immune cell populations [18,19]. At specific loci implicated in lupus, including *IRF5* and *TNFAIP3*, functional variants have been shown to alter transcription factor binding and downstream inflammatory gene expression, providing direct experimental evidence that noncoding variation modulates immune signaling pathways rather than protein structure [20,21,22].

Among the strongest genetic signals are variants within the major histocompatibility complex (MHC). Specific HLA-DR and HLA-DQ alleles influence peptide presentation and shape autoreactive T cell repertoires, thereby affecting downstream autoantibody responses [1]. Although the precise mechanisms remain incompletely resolved, fine-mapping studies indicate that multiple independent variants within the MHC region contribute to disease susceptibility.

Outside the MHC, several loci influence signaling pathways that regulate innate and adaptive immune activation. Polymorphisms in transcription factors such as IRF5 and STAT4 are consistently associated with SLE across populations and can alter transcriptional response downstream of nucleic acid sensing pathways and cytokine receptors, thereby modulating inflammatory gene expression programs in immune cells [2]. Genetic variation in B cell signaling molecules, including BLK and BANK1, affects B cell receptor signaling thresholds and may influence the survival and differentiation of autoreactive B cell populations [23]. Variants affecting regulatory proteins that terminate inflammatory signaling also contribute to susceptibility. For example, polymorphisms in *TNFAIP3*, encoding the ubiquitin-editing enzyme A20, impair negative regulation of NF-κB signaling and are associated with increased inflammatory responses in immune cells and experimental models [12]. Representative polymorphisms and their functional roles are summarized in Table 1.

Rare monogenic disorders with lupus-like phenotypes have provided additional insight into pathways that restrain immune activation. Loss-of-function mutations in genes involved in nucleic acid metabolism, including *TREX1*, *RNASEH2*, and *DNASE1L3*, lead to accumulation of endogenous nucleic acid species capable of activating innate immune sensors (Table 1).

Studies in patients and genetically engineered mice demonstrate that these defects trigger chronic inflammatory responses and systemic autoimmunity resembling lupus [8,28,29]. Although such mutations account for only a small fraction of SLE cases, they highlight biological pathways that are relevant to the broader disease.

Interpretation of lupus risk loci has increasingly relied on integrative genomic approaches. Expression quantitative trait locus analyses indicate that many susceptibility variants influence gene expression in a cell-type-specific manner, particularly in B cells, T cells, and monocytes [4]. Chromatin accessibility mapping further shows that a substantial proportion of lupus-associated variants reside within enhancer elements responsive to immune activation signals [2]. These findings support a model in which genetic variants alter regulatory circuitry that controls immune gene expression rather than producing isolated molecular defects.

Structural genomic variation also contributes to disease risk. Copy number variation at the complement component C4 locus has emerged as one of the strongest genetic determinants of SLE susceptibility. Individuals with reduced C4A gene copy numbers exhibit impaired clearance of immune complexes and apoptotic debris, processes that are important for maintaining immune tolerance [27]. In addition, sex chromosome biology contributes to disease susceptibility. Incomplete X-chromosome inactivation of immune-related genes, including *TLR7*, has been proposed as a mechanism that increases immune gene dosage in females and may contribute to the pronounced sex bias observed in SLE [1]. The combination of multiple low-effect common variants, rare coding variants, and structural copy number differences establishes a complex, multilayered genetic framework that interacts with epigenetic modifications and environmental triggers to shape the trajectory of disease.

The emerging view is that SLE does not arise from a simple linear cascade of isolated defects. Instead, it reflects a networked system in which genetic variation primes the immune landscape, sensitizes nucleic acid sensors, amplifies interferon responses, and predisposes adaptive immune cells to pathogenic activation. Experimental studies in murine models, including *SCARF1*-deficient mice, further illustrate how impaired ACs clearance interacts with genetic susceptibility to accelerate autoimmunity, demonstrating the necessity of considering both inherited and context-dependent molecular mechanisms [30]. This network perspective, integrating population-level genetics, single-cell regulatory mapping, and mechanistic studies, provides a foundational layer for understanding SLE as a system-wide dysregulation of immune tolerance, setting the stage for mechanistic and translational insights into epigenetic regulation, environmental modulation, and downstream cellular reprograming.

## 3. Epigenetic Regulation in Systemic Lupus Erythematosus

Epigenetic mechanisms represent a critical regulatory layer linking genetic susceptibility to altered immune cell behavior in SLE. Epigenetic modifications influence gene expression without altering the underlying DNA sequence and include processes such as DNA methylation, histone modification, and regulation by noncoding RNAs. Accumulating evidence indicates that abnormalities in these regulatory systems contribute to immune dysregulation observed in lupus [31,32].

Alterations in DNA methylation represent one of the most extensively studied epigenetic features of SLE [33]. Early investigations demonstrated that CD4^+^ T cells from patients exhibit global hypomethylation relative to cells from healthy individuals [34,35]. Subsequent studies identified specific loci in which promoter demethylation leads to increased expression of immune activation genes. For example, demethylation of regulatory elements controlling CD40L expression on T cells results in enhanced CD40L surface expression and promotes B cell activation in vitro [36]. Similarly, hypomethylation of genes such as ITGAL and PRF1 has been associated with altered T cell activation states and increased cytotoxic potential [37]. Experimental models further demonstrate that pharmacologic inhibition of DNA methyltransferases can induce lupus-like immune phenotypes in mice, suggesting that disruption of methylation homeostasis may contribute to autoimmune pathology [32].

Histone modifications shape the transcriptional landscape of immune cells in lupus. Chromatin immunoprecipitation studies have identified enrichment of activating histone marks such as H3K4me3 and H3K27ac at promoters of *IFN*-responsive genes in immune cells derived from patients with active disease [38]. These modifications promote open chromatin configurations that facilitate transcriptional activation. In contrast, reductions in repressive histone marks, including H3K9me3 and H3K27me3, have been observed at several inflammatory gene loci. This data suggests that loss of transcriptional repression may contribute to persistent immune activation [32].

In addition to histone modifications, noncoding RNAs (ncRNAs) represent another important layer of epigenetic regulation that influences gene expression without encoding proteins. Noncoding RNAs can regulate transcriptional programs through interactions with chromatin-modifying complexes, modulation of transcription factors, and post-transcriptional control of messenger RNAs. Among these, microRNAs regulate gene expression by targeting messenger RNAs for degradation or translational repression [39]. Several microRNAs have been implicated in lupus pathogenesis [40,41,42]. For example, reduced expression of miR-146a in immune cells from patients has been associated with enhanced activation of inflammatory signaling pathways and increased production of cytokines [1]. Other microRNAs, including miR-155 and miR-21, influence differentiation and activation of lymphocytes and have been shown to modulate immune responses in experimental models of autoimmunity [43]. Another important class of ncRNAs is long noncoding RNAs (lncRNAs), which are transcripts typically longer than 200 nucleotides. lncRNAs contribute to epigenetic regulation by interacting with chromatin-modifying complexes and influencing transcriptional activity at specific genomic loci. Several lncRNAs have been reported to be differentially expressed in lupus immune cells, although their functional roles remain an area of active investigation [32].

Genome-wide epigenomic analyses provide further insight into the relationship between genetic susceptibility loci and chromatin regulation. Many genomic regions associated with lupus risk overlap with regulatory elements that display altered chromatin accessibility in immune cells from patients [2]. These findings suggest that inherited variants and epigenetic modifications may converge on shared regulatory regions to influence immune gene expression. Single-cell epigenomic studies have begun to reveal cell-type-specific chromatin alterations in lupus immune populations, indicating that epigenetic dysregulation may affect distinct immune compartments in different ways [2].

**Figure 2:**
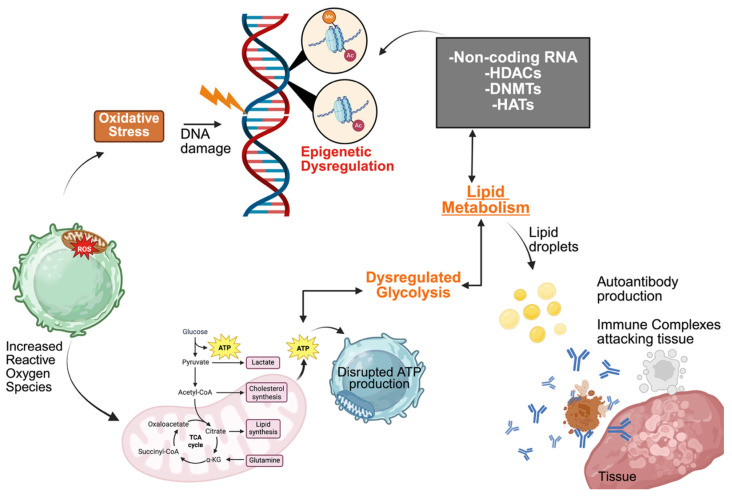
Epigenetic and metabolic alterations drive autoimmune pathogenesis. This drawing depicts the interconnected epigenetic and metabolic changes contributing to autoimmune disease. Oxidative stress, marked by increased reactive oxygen species (ROS), causes DNA damage, initiating epigenetic dysregulation through mechanisms involving noncoding RNA, histone deacetylases (HDACs), DNA methyltransferases (DNMTs), and histone acetyltransferases (HATs). These epigenetic changes impact both lipid metabolism and glycolysis. Dysregulated glycolysis leads to disrupted ATP production and altered cellular energy processes, while changes in lipid metabolism result in the formation of lipid droplets. Together, these metabolic alterations promote immune dysfunction, including autoantibody production and immune complexes attacking tissue, which culminate in autoimmune pathology. Arrows indicate the sequence and interplay between these pathways. https://BioRender.com/qehher6 (accessed on 15 April 2026).

Collectively, epigenetic dysregulation in SLE represents both a consequence and amplifier of immune dysfunction. Changes in DNA methylation, histone modifications, and noncoding RNA expression collectively influence transcriptional programs that regulate immune activation and tolerance (Figure 2). Understanding this multilayered regulation provides a mechanistic bridge linking genetic predisposition to phenotypic heterogeneity and highlights potential avenues for epigenetic therapies aimed at restoring chromatin homeostasis, attenuating interferon-driven inflammation, and normalizing immune cell function.

## 4. Environmental and Hormonal Influences in Systemic Lupus Erythematosus

Environmental and hormonal stressors are critical factors that shape susceptibility to SLE and modulate disease activity in genetically predisposed individuals. Epidemiologic and experimental studies indicate that environmental exposures can precipitate disease onset or exacerbate established autoimmunity [44]. However, these factors rarely act in isolation; instead, they interact with underlying genetic and epigenetic susceptibility. Among the most consistently implicated environmental triggers are ultraviolet (UV) radiation, viral infection, cigarette smoking, and certain occupational or chemical exposures. These factors influence immune regulation through mechanisms that include induction of cell death, modification of self-antigens, and activation of innate immune pathways, thereby creating conditions that may promote loss of tolerance [45].

Ultraviolet radiation is among the best-characterized environmental contributors to lupus flares, particularly in patients with cutaneous disease. Ultraviolet B (UVB) exposure induces cell death in keratinocytes, leading to the release and surface translocation of nuclear autoantigens such as Ro, La, and nucleosomal components [46,47]. Furthermore, UV irradiation promotes clustering of these nuclear and nucleosomal autoantigens on apoptotic blebs, which can be recognized by autoantibodies present in lupus sera [48]. In addition to promoting autoantigen availability, UV radiation stimulates local production of inflammatory cytokines and chemokines in the skin, facilitating recruitment of immune cells and amplification of inflammatory responses [49]. A study by Furukawa et al. demonstrated in lupus-prone MRL/lpr mice that UV-B irradiation exacerbated cutaneous disease with increased inflammatory infiltration and disease severity compared with unexposed controls [50]. These findings suggest that UV radiation contributes to disease exacerbation primarily by altering antigen availability and inflammatory signaling rather than acting as an independent initiating factor.

Infectious agents have been implicated in lupus pathogenesis, with the strongest epidemiologic and mechanistic evidence involving Epstein–Barr virus (EBV) [51]. Serologic studies consistently demonstrate higher titers of antibodies against EBV antigens in patients with lupus compared with healthy controls [1]. James and colleagues reported markedly elevated antibody responses to EBV viral capsid antigen and EBV nuclear antigen-1 in individuals with systemic lupus erythematosus compared with healthy controls [52].

EBV infects B lymphocytes and establishes latent infection, providing a potential reservoir of viral antigens capable of sustaining immune activation. Molecular mimicry between EBV proteins and host autoantigens has been proposed as one mechanism contributing to autoantibody development [53,54,55]. For example, sequence homology between EBV nuclear antigen 1 and lupus autoantigens has been shown to induce cross-reactive antibody responses in experimental systems [1]. Additional mechanistic evidence was provided by Poole BD and colleagues, who demonstrated cross-reactivity between antibodies generated against EBV nuclear antigen-1 and lupus autoantigens in experimental models [54]. Furthermore, EBV infection can influence B cell differentiation and survival pathways, processes that are already dysregulated in lupus [1]. Although EBV infection is nearly ubiquitous in the general population, these findings suggest that host susceptibility factors determine whether viral exposure contributes to autoimmune activation.

Environmental toxins and lifestyle-related exposures are associated with increased lupus risk or disease severity. Cigarette smoking is among the most reproducibly identified risk factors. Epidemiologic studies indicate that current smokers have a higher likelihood of developing SLE, with severe disease manifestations compared with non-smokers [45]. Mechanistically, cigarette smoke contains numerous oxidizing agents capable of inducing oxidative stress and modifying proteins and lipids, potentially generating neoantigens that stimulate immune responses [44]. Smoking has also been shown to influence immune cell function, including altered cytokine production and changes in antigen-presenting cell activity [56]. While these mechanisms remain incompletely defined in lupus specifically, the cumulative evidence supports smoking as a modifiable environmental risk factor that may amplify inflammatory processes in susceptible individuals.

Hormonal influences are another major determinant of lupus susceptibility and clinical expression. The marked female predominance of SLE, with a 9:1 female-to-male ratio during reproductive years, suggests an important role for sex hormones in modulating immune responses [57]. Estrogens exert diverse immunomodulatory effects, including enhancement of B cell maturation and survival and modulation of cytokine production by T lymphocytes and antigen-presenting cells. Experimental studies have shown that estrogen signaling can influence expression of activation-induced cytidine deaminase and other molecules involved in antibody diversification, thereby affecting humoral immune responses [58]. One study using the lupus-prone murine strains NZB/W F1 mice, the researchers discovered that estrogen administration accelerates disease progression, whereas ovariectomy delays onset of nephritis and reduces autoantibody production [57]. Additional reports have demonstrated that estradiol promotes survival of autoreactive B cells and increases anti-double-stranded DNA antibody production by interfering with B cell tolerance mechanisms [59]. Furthermore, estrogen receptor signaling directly alters gene expression programs in B cells, including upregulation of survival molecules such as Bcl-2, thereby promoting autoreactive B cell persistence [60]. These findings indicate that estrogen can modulate disease expression in genetically susceptible hosts.

In contrast to estrogens, androgens exert an immunosuppressive effect in many experimental systems. Testosterone has been shown to reduce B cell activation and dampen inflammatory cytokine production in murine lupus models [57]. Male lupus-prone mice treated with testosterone display delayed disease onset and reduced severity of renal pathology, suggesting that androgen signaling may counterbalance proinflammatory immune pathways. Progesterone has also been reported to influence immune responses by promoting regulatory T cell differentiation and limiting inflammatory T helper cell responses, although the relevance of these effects in human lupus remains less clearly defined [57,58]. Moreover, vitamin D, which acts as a steroid hormone, functions as an immune modulator that can suppress inflammation, improve vascular health, and regulate T-cell function, potentially helping to alleviate SLE symptoms [61].

Beyond individual environmental or hormonal exposures, emerging evidence indicates that additional biological variables including circadian regulation, microbial colonization, and nutritional status can influence immune homeostasis and potentially modify autoimmune susceptibility [2]. For example, alterations in gut microbiota composition have been observed in lupus-prone mice and in patients with SLE, and experimental manipulation of microbial communities can influence disease severity in animal models [2]. While the data is complicated, alterations in the microbiota have been shown to correlate with SLE [62,63,64]. However, nutritional factors and metabolic state affect the immune responses through effects on cellular metabolism and inflammatory signaling pathways, although their precise contribution to lupus pathogenesis remains an active area of investigation.

Experimental systems integrating environmental triggers with genetic susceptibility highlight the multifactorial nature of lupus development. Lupus-prone mouse strains exposed to UV radiation, viral mimetics, or other environmental stressors frequently display accelerated disease progression compared with unexposed controls, including earlier autoantibody production and increased organ pathology [65]. Such findings support the concept that environmental exposures may modify the trajectory of disease in genetically susceptible hosts rather than acting as universal causes of lupus.

In summary, environmental and hormonal influences represent important modulators of lupus susceptibility and disease activity. Exposure to UV radiation, viral infection, smoking, and hormonal signaling can alter immune responses through effects on antigen availability, inflammatory signaling pathways, and immune cell function. These factors interact with genetic and epigenetic predispositions to shape the timing and severity of autoimmune manifestations. Understanding these interactions provides insight into why disease onset and flares often follow environmental exposures and highlights the importance for personalized intervention strategies, such as lifestyle modification, viral prophylaxis, and targeted hormonal modulation, which could complement immunomodulatory therapies and improve patient outcomes.

## 5. Nucleic Acid Sensing and Interferon Signaling

Recognition of nucleic acids by innate immune receptors represents a fundamental component of host defense against viral and microbial pathogens. In SLE, however, this protective system becomes inappropriately engaged by endogenous nucleic acids derived from apoptotic or damaged cells, leading to sustained activation of interferon-dependent inflammatory pathways [66]. A large body of clinical and experimental evidence indicates that type I IFN signaling is a major immunologic feature of SLE, as reflected by the elevated expression of IFN-stimulated genes observed in peripheral blood and affected tissues of patients [8,67,68]. Rather than representing a single initiating event, aberrant nucleic acid recognition appears to function as an amplifying mechanism that sustains immune activation once tolerance to nuclear antigens has been breached.

Plasmacytoid dendritic cells (pDCs) are a principal source of type I IFN in lupus. pDCs express high levels of endosomal Toll-like receptors capable of detecting nucleic acids, particularly Toll-like receptor (TLR) 7 (TLR7), which recognizes single-stranded RNA, and Toll-like receptor 9 (TLR9), which senses unmethylated CpG-rich DNA motifs. Immune complexes containing nucleic acids and autoantibodies can be internalized by Fcγ receptor-mediated uptake and delivered to endosomal compartments, where they activate TLR signaling pathways [69,70]. Activation of these receptors induces downstream signaling through adaptor molecules such as MyD88 and transcription factors, including IRF7, culminating in robust production of type I IFN. In vitro studies using human pDCs demonstrate that immune complexes isolated from lupus patient sera can induce strong interferon responses, supporting the concept that nucleic-acid-containing immune complexes act as endogenous ligands for these receptors [71].

Genetic studies support the importance of nucleic acid sensing pathways in lupus susceptibility. Variants in genes encoding components of interferon signaling and nucleic acid recognition pathways, including IRF5, IRF7, and TLR7, have been reproducibly associated with increased disease risk in GWAS studies [2,23]. Functional analyses indicate that some of these variants influence transcriptional responses downstream of Toll-like receptor activation, thereby modulating the magnitude of interferon production. In addition, rare mutations affecting enzymes responsible for degrading endogenous nucleic acids highlight the importance of nucleic acid homeostasis in preventing autoimmunity [72]. Loss-of-function mutations in the exonuclease TREX1 lead to accumulation of cytosolic DNA and are associated with inflammatory syndromes that include lupus-like manifestations [2,8]. Mouse models lacking TREX1 develop systemic inflammation driven by activation of cytosolic DNA sensing pathways, illustrating how impaired clearance of nucleic acids can provoke interferon-mediated immune activation [73].

Cytosolic nucleic acid sensors contribute to IFN responses in lupus. The cyclic GMP-AMP synthase (cGAS)-STING pathway detects double-stranded DNA within the cytoplasm and induces production of type I interferons through activation of TBK1 and IRF3 signaling cascades [74]. A study by Ablasser et al. suggested that accumulation of cytosolic DNA in the absence of nucleases such as TREX1 can activate this pathway, leading to chronic interferon production and inflammatory disease in mice [75]. Although the precise contribution of cGAS-STING signaling to human SLE remains under active investigation, elevated activity of this pathway has been reported in several experimental models of nucleic-acid-driven autoimmunity. Together, endosomal (TLR7/9) and cytosolic (cGAS-STING and RIG I like receptor) pathways coordinate detection of nucleic acids across cellular compartments.

In addition to DNA sensing via cGAS-STING, cytosolic RNA sensing pathways mediated by retinoic-acid-inducible gene I (RIG I; DDX58) and melanoma differentiation-associated protein 5 (MDA5; IFIH1) contribute to type I interferon induction. These RIG I like receptors (RLRs) detect viral and endogenous double-stranded or 5′-triphosphorylated RNA and signal through the mitochondrial adaptor MAVS, leading to activation of IRF3 and NF-κB and subsequent type I IFN production [76,77,78]. Experimental studies have demonstrated that engagement of RLR pathways can drive robust interferon responses comparable to DNA-sensing pathways under conditions of cellular stress or RNA accumulation [79,80]. Genetic association studies in SLE further support a contributory role of this axis, as variants in IFIH1 (encoding MDA5) have been linked to altered interferon signaling and autoimmune susceptibility in multiple cohorts [4]. Although their contribution in SLE is less extensively characterized than TLR7/9 or cGAS–STING pathways, RLR signaling represents an additional cytosolic nucleic-acid-sensing mechanism capable of amplifying interferon-driven inflammation in contexts of endogenous RNA release and impaired nucleic acid clearance [8].

Type I IFN exerts wide-ranging effects on both innate and adaptive immune responses. These cytokines promote maturation of dendritic cells (DCs), enhance antigen presentation, and influence the differentiation of multiple lymphocyte populations [81]. Exposure of B cells to type I IFN increases expression of activation markers and can enhance responsiveness to antigen receptor stimulation. Interferon signaling also affects T lymphocyte function, influencing cytokine production and shaping helper T cell differentiation [82]. Through these mechanisms, sustained interferon activity can create an immunologic environment that favors persistence of autoreactive immune responses. Studies in lupus-prone mice lacking the type I IFN receptor demonstrate reduced autoantibody production and attenuated renal disease, providing experimental evidence that interferon signaling contributes to disease progression in these models [68].

Recent work has revealed additional regulatory layers that influence nucleic-acid-induced interferon responses. Post-transcriptional control mechanisms, including RNA-binding proteins, modulate the stability and translation of cytokine transcripts involved in innate immune signaling. The cellular nucleic-acid-binding protein (CNBP) has been shown to regulate transcriptional responses to microbial and endogenous nucleic acids through effects on inflammatory gene expression pathways [83]. Studies using CNBP-deficient mice indicate that disruption of this regulatory system alters cytokine production and immune cell activation following nucleic acid stimulation, suggesting that RNA-binding proteins contribute to fine-tuning innate immune responses.

**Figure 3:**
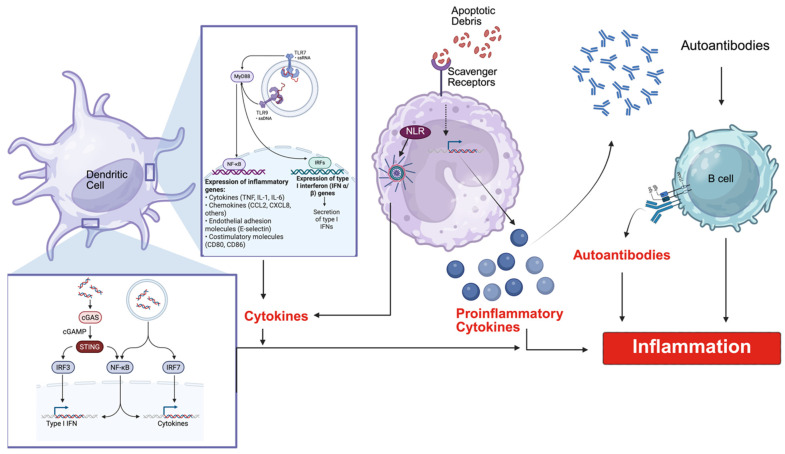
Molecular circuits linking innate immune sensing to chronic inflammation in autoimmunity. This figure outlines the interconnected molecular and cellular pathways that drive chronic inflammation in autoimmune disease. Dendritic cells serve as key sensors of nucleic acids derived from apoptotic debris or damaged tissue. Internal pattern recognition receptors, including Toll-like receptors (TLR7 for ssRNA and TLR9 for dsDNA) and the cGAS-STING pathway, engage adaptor proteins such as MyD88 and STING to transduce signals that activate transcription factors IRF3, IRF7, and NF-κB. This results in increased expression of proinflammatory genes encoding cytokines (e.g., TNFα and IL-6), chemokines, adhesion molecules, and type I interferons. Phagocytes detect apoptotic debris through scavenger receptors and cytosolic sensor proteins (NLRs), importing nucleic acids and further amplifying cytokine production. Released proinflammatory cytokines create a feed-forward loop, recruiting and activating additional immune cells and influencing B cell function. Activated B cells mount an autoimmune response characterized by production of pathogenic autoantibodies, which form immune complexes that deposit in tissues, triggering complement activation, cellular infiltration, and sustained injury. Insets highlight detailed nucleic acid sensing pathways—TLR and STING signaling—leading to expression of costimulatory molecules and cytokines that perpetuate the inflammatory environment. Arrows map the sequential and recursive interactions between innate sensing, cytokine signaling, autoantibody production, and downstream tissue damage, emphasizing their collective role in chronic autoimmune inflammation. Arrows indicate directionality of the process from each immune cell type. https://BioRender.com/011ioan (accessed on 15 April 2026).

Clearance mechanisms that regulate the availability of extracellular nucleic acids influence activation of innate immune signaling cascade pathways (Figure 3). Phagocytic receptors and scavenger receptors (SR) expressed on macrophages and DCs participate in the uptake of apoptotic material and nucleic-acid-containing immune complexes. Studies have shown that defects in clearance pathways can increase the persistence of extracellular nucleic acids and immune complexes, thereby facilitating their recognition by Toll-like receptors within endosomal compartments [30,84,85]. These findings suggest that impaired removal of cellular debris may indirectly enhance activation of nucleic acid sensing pathways in lupus.

Environmental factors can further modulate IFN responses by increasing the availability of immunostimulatory nucleic acids. Viral infections and UV radiation can induce cell death and release nucleic acids that activate innate immune receptors. Experimental models demonstrate that exposure to viral nucleic acid mimetics such as poly(I:C) or CpG oligonucleotides enhances interferon responses and can accelerate disease manifestations in lupus-prone mice [69]. These observations provide a mechanistic explanation for clinical observations in which infections or tissue injury are associated with disease flares.

Therapeutic strategies targeting interferon signaling pathways have attracted considerable interest. Neutralizing antibodies directed against IFN-α or the type I IFN receptor have been developed to interrupt this signaling axis. Clinical trials evaluating blockade of interferon receptor signaling have demonstrated reductions in interferon-stimulated gene expression and improvements in disease activity in subsets of patients with SLE [86,87]. Although not universally effective, these results provide clinical evidence that IFN signaling contributes to disease activity in at least a proportion of patients. Thus, genetic susceptibility, defects in nucleic acid clearance, and environmental exposures can all influence the magnitude of these responses, illustrating how multiple pathogenic mechanisms converge on interferon signaling pathways in lupus. Intercepting the IFN-driven feed-forward loop that underlies SLE could provide a framework for precision therapeutics, highlighting both mechanistic insight and clinical opportunity.

## 6. B Cells and Autoantibody Production

B lymphocytes play a central role in the immunopathogenesis of SLE. These lymphocytes function not only as producers of pathogenic autoantibodies but also as antigen-presenting cells and regulators of cytokine networks that shape adaptive immune responses [1,88]. Multiple checkpoints normally constrain autoreactive B cells during development; however, in SLE these mechanisms become ineffective, allowing autoreactive clones to persist and expand [89]. Central tolerance mechanisms in the bone marrow eliminate or edit many self-reactive B cells, yet studies using single-cell B cell receptor sequencing demonstrate that autoreactive specificities remain detectable among peripheral naïve B cells in patients with SLE, suggesting incomplete negative selection [2,44]. Peripheral tolerance mechanisms, including anergy, receptor editing, and regulatory cell-mediated suppression, further limit autoreactivity under physiological conditions but, in lupus, these safeguards become destabilized, permitting autoreactive B cells to participate in immune activation and differentiation.

A defining immunologic feature of SLE is the production of high-affinity autoantibodies directed against nuclear antigens including dsDNA, nucleosomes, and small ribonucleoprotein complexes [90]. These antibodies arise largely from germinal center reactions in secondary lymphoid organs, where somatic hypermutation and affinity maturation occur. Histologic and transcriptomic analyses of lupus-prone mouse strains and patient lymphoid tissues demonstrate persistent or expanded germinal center structures associated with autoreactive B cell clones [2,91]. Disruption of germinal center formation through genetic deletion of key regulatory molecules such as Bcl6 or ICOS in murine lupus models markedly reduces anti-dsDNA antibody production and ameliorates renal pathology, supporting the mechanistic importance of germinal center dynamics in sustaining humoral autoimmunity [2].

Signals derived from innate immune receptors strongly influence B cell activation thresholds in lupus. Immune complexes containing nucleic acids can engage B cell receptors while simultaneously activating endosomal TLR, particularly TLR7 and TLR9, leading to synergistic activation signals that promote proliferation and plasmablast differentiation [92]. It was shown that B cells from lupus-prone mice respond vigorously to RNA-containing immune complexes through TLR7-dependent pathways, producing proinflammatory cytokines and differentiating into antibody-secreting cells [84,93]. Genetic deletion or pharmacologic inhibition of TLR7 significantly reduces autoantibody production and glomerulonephritis in murine lupus models, whereas increased TLR7 gene dosage accelerates disease, highlighting the pathogenic potential of nucleic-acid-sensing pathways in shaping B cell responses [2].

Transcriptional programs regulate the differentiation of B cells into antibody-secreting plasma cells. Transcription factors, including IRF4, BLIMP-1 (encoded by PRDM1), and XBP1, coordinate plasma cell differentiation and immunoglobulin secretion [94]. Studies in lupus-prone mice indicate that dysregulated expression of these transcriptional regulators can enhance plasmablast expansion and antibody production [2]. Conversely, perturbation of these pathways through genetic manipulation or targeted inhibition reduces plasmablast formation and attenuates disease manifestations in experimental models, emphasizing the importance of transcriptional control in autoreactive B cell fate decisions.

Autoantibodies exert pathogenic effects primarily through the formation of immune complexes that deposit in tissues and trigger inflammatory cascades. These complexes activate complement pathways and engage Fcγ receptors on myeloid cells, resulting in cytokine release, leukocyte recruitment, and local tissue injury [95]. Classic studies in murine lupus models (NZB/NZW F1) demonstrate that deficiency of activating Fcγ receptors significantly attenuate nephritis despite continued autoantibody production, illustrating that immune-complex-mediated effector pathways are critical determinants of tissue pathology [2,96]. Similarly, complement components such as C3 and C4 influence immune complex clearance and inflammatory signaling, and their deficiency or dysregulation alters susceptibility to lupus-like disease [97].

B cells also shape immune responses through cytokine production and antigen presentation. Activated B cells can produce cytokines, including IL-6, TNF, and IL-10, thereby influencing T cell differentiation and inflammatory responses [2,98]. Experimental depletion of B cells in lupus-prone mice reduces T cell activation and inflammatory cytokine production even in the absence of immediate changes in autoantibody titers, demonstrating that B cells contribute to disease pathogenesis through antibody-independent mechanisms [2].

Mechanistic insights into these pathogenic B cell activities, encompassing autoantibody secretion, antigen presentation, and cytokine production have guided therapeutic strategies targeting B cells in SLE. Monoclonal antibodies directed against CD20 deplete circulating B cells, whereas inhibitors of B cell survival factors such as BAFF modulate the maturation and persistence of autoreactive clones [99]. Clinical trials evaluating BAFF inhibition show a reduction in disease activity and autoantibody titers in subsets of patients with lupus, confirming that perturbation of B cell survival pathways can alter disease trajectories [2,100]. Together, these observations support a model in which B cell dysregulation arises from the convergence of defective tolerance checkpoints, amplified innate receptor signaling, and transcriptional programs that favor plasma cell differentiation. By integrating antigen presentation, cytokine production, and autoantibody secretion, B cells act as central orchestrators of adaptive immune dysfunction in SLE.

## 7. T Cell Dysregulation

T lymphocytes play a critical role in shaping autoimmune responses in SLE, and multiple abnormalities in T cell signaling, differentiation, and regulatory capacity have been documented in both patients and experimental models. CD4^+^ helper T cells and CD8^+^ cytotoxic T cells exhibit altered functional phenotypes that contribute to persistent immune activation and impaired tolerance [101]. One of the most consistently observed alterations in SLE involves skewing of CD4^+^ T cell differentiation toward proinflammatory subsets, including T helper 1 (Th1), T helper 17 (Th17), and T follicular helper (Tfh) cells, accompanied by instability or functional impairment of regulatory T cell populations [1,102]. The shift in T cell subset balance creates an immunologic environment that favors B cell activation and chronic inflammation.

Abnormalities in T cell receptor signaling pathways represent a hallmark of lupus T cell dysfunction. Peripheral T cells from patients with SLE exhibit alterations in signaling intermediates downstream of the T cell receptor complex, including reduced expression of the CD3-zeta chain and compensatory signaling through Fc receptor γ chains [103]. This molecular rewiring enhances calcium signaling and alters activation thresholds, resulting in exaggerated responses to antigenic stimulation [2]. Enhanced calcium flux and downstream activation of transcription factors such as NFAT promote increased cytokine production and proliferation, thereby facilitating the expansion of autoreactive T cell clones.

Importantly, these disruptions in signaling are closely tied to changes in the structural organization of the T cell membrane, particularly the lipid rafts that orchestrate receptor signaling events. Lipid rafts are essential in the regulation of T-lymphocyte activation pathways [104]. In normal T cells, T cell receptor (TCR) engagement triggers the rapid aggregation of lipid rafts resulting in the accumulation of signaling proteins at the immunological synapse. They serve as critical platforms for the assembly of the TCR complex with downstream signaling proteins such as Lck, LAT, and ZAP-70. Upon antigen recognition, lipid rafts facilitate phosphorylation cascades by concentrating these kinases and adaptors at the immunological synapse, amplifying TCR signaling [105]. However, in lupus T cells membrane morphology and composition is altered, with an increase in lipid rafts that are already clustered [106]. Studies have identified that changes in cholesterol and sphingolipids content can lead to enhanced T cell activation by lowering the threshold for activation and promoting auto-reactive T cell responses [107]. McDonald et al. showed a profound alteration on glycosphingolipids in CD4^+^ T cells from SLE patients, and normalizing their expression could lead to the rectification of the alterations [108]. These findings support the model that dysregulated lipid raft composition can modulate TCR thresholds and promote autoimmunity. This leads to impaired immune tolerance and inflammation, a core defect in SLE [104,109,110,111]. While lipid raft alterations affect global T cell activation, it is also important to consider how specific CD4^+^ T cell subsets, such as T follicular helper (Tfh) cells, contribute uniquely to lupus pathogenesis.

Among CD4^+^ T cell subsets, Tfh cells are particularly important in lupus pathogenesis because of their role in supporting germinal center responses and antibody production [112]. Increased frequencies of circulating Tfh-like cells have been reported in patients with active SLE and correlate with disease severity and autoantibody titers [113,114]. These cells express high levels of ICOS, PD-1, and the transcription factor Bcl6, and they produce cytokines such as IL-21 that promote B cell proliferation and differentiation. Studies in lupus-prone mouse models show that disruption of Tfh differentiation pathways reduces germinal center formation and attenuates autoantibody production, highlighting the critical contribution of Tfh-B cell interactions to disease progression [115].

The role of CD8^+^ T cells in lupus is complex and varies across disease stages and tissues. Some studies report impaired cytotoxic function in circulating CD8^+^ T cells from patients with SLE, potentially contributing to defective clearance of autoreactive immune cells or infected targets [1,116,117]. However, other investigations have identified expanded populations of activated cytotoxic T cells in inflamed tissues, including the kidneys of patients with lupus nephritis, where they may contribute to local tissue injury through perforin- and granzyme-mediated mechanisms [2]. These findings suggest that CD8^+^ T cell responses in lupus are heterogeneous and influenced by the inflammatory microenvironment.

Chronic inflammatory signaling in systemic lupus erythematosus (SLE) has been associated with increased expression of inhibitory receptors on peripheral T cells, consistent with an “exhaustion-like” or dysfunctional activation state rather than canonical T cell exhaustion. In peripheral T cells from patients with SLE, programed cell death-1 (PD-1) expression is significantly increased compared with healthy controls and has been linked to disease activity and altered immune regulation [118]. Similarly, expression of the inhibitory receptor Tim-3 (HAVCR2) has been reported on peripheral T cells in SLE and is associated with immune dysregulation and disease activity [119]. Functionally, SLE T cells exhibit impaired activation responses, including reduced IL-2 production and abnormal TCR signaling thresholds, which contribute to diminished proliferative capacity and altered effector differentiation [107]. In addition to inhibitory receptor expression, SLE T cells display features of chronic activation and altered differentiation states, particularly within CD8^+^ T cell compartments. Persistent inflammatory and antigenic stimulation in SLE is associated with a shift toward highly differentiated T cell phenotypes with reduced functional responsiveness [120]. These phenotypic and functional characteristics align with broader immunological models in which chronic antigen exposure induces inhibitory receptor programs and progressive loss of effector function, as described in canonical T cell exhaustion frameworks [121]. Similar dysfunctional T cell states arising from sustained immune stimulation have also been described in chronic immune activation contexts and provide a conceptual framework for interpreting T cell dysregulation in SLE [122]. Collectively, these findings support a model in which chronic inflammation in SLE is associated with inhibitory receptor expression and functional impairment in T cells, alongside altered differentiation toward long-lived, hyporesponsive effector states.

Another critical component of T cell homeostasis that is perturbed in lupus is the regulatory T-cell compartment (Tregs). Multiple studies have reported reduced frequencies or impaired suppressive function of Foxp3-expressing Tregs in patients with SLE [2]. Experimental expansion or adoptive transfer of Tregs in lupus-prone mouse models suppresses autoantibody production and attenuates tissue inflammation, indicating that restoration of regulatory networks can modulate disease severity [2]. Mechanistically, inflammatory cytokines such as IL-6 and type I interferons can destabilize Foxp3 expression and impair Tregs function, thereby permitting the expansion of autoreactive effector populations.

In lupus T cell behavior is shaped by metabolic and transcriptional reprograming. SLE T cells exhibit alterations in mitochondrial function and increased reliance on glycolytic metabolism, which supports sustained activation and cytokine production [123]. Transcription factors, including STAT1, STAT3, and Tβ, regulate the differentiation of inflammatory T cell subsets, whereas dysregulated signaling through these pathways has been observed in both human disease and experimental models [51,124]. Pharmacologic interventions targeting metabolic pathways, including inhibition of mTOR signaling, have demonstrated the ability to normalize T cell activation and reduce disease activity in experimental lupus and early clinical studies [125,126]. Through direct effector functions and by providing help to autoreactive B cells, dysregulated T cells contribute substantially to the amplification and persistence of systemic autoimmunity. Therapeutic strategies aimed at restoring T cell homeostasis, including modulation of costimulatory pathways, cytokine signaling, and metabolic programs, therefore represent promising avenues for limiting immune activation and preventing tissue damage in lupus.

## 8. Scavenger Receptors and Clearance Pathways in Lupus

Efficient removal of ACs and cellular debris is a fundamental homeostatic process that prevents the accumulation of immunogenic self-antigens and limits activation of innate immune pathways [127]. Failure of this process is associated with SLE, where persistent apoptotic material and immune complexes provide a continuous source of endogenous danger signals [28]. Among the molecular systems responsible for debris clearance, SRs represent a broad family of pattern-recognition receptors expressed on macrophages, DCs, endothelial cells, and certain epithelial populations. These receptors bind diverse ligands, including oxidized lipoproteins, apoptotic membranes, nucleic acid–protein complexes, and modified self-antigens, thereby facilitating phagocytic uptake and intracellular degradation [128,129,130]. Dysregulation of these clearance pathways has been proposed to contribute to lupus pathogenesis by allowing persistence of immunostimulatory material capable of activating innate immune sensors.

Multiple members of the SRs family have been implicated in the recognition and uptake of ACs. Class A scavenger receptors such as SR-A1 (encoded by *MSR1*) and MARCO bind polyanionic ligands and microbial or endogenous debris, promoting phagocytic uptake by macrophages [131,132,133]. Murine models show that deficiency of these receptors alters the efficiency of ACs removal and modifies inflammatory responses to cellular debris, although the precise contribution of each receptor to lupus pathogenesis remains complex and context-dependent [134,135]. Rather than acting as isolated determinants of disease, these receptors appear to function within redundant and cooperative networks of phagocytic receptors that collectively regulate clearance efficiency.

Members of the class B scavenger receptor family also participate in the recognition of modified lipids and apoptotic membranes. CD36 and SCARB1 (SR-BI) interact with oxidized phospholipids and ACs-derived lipids, contributing to macrophage uptake of dying cells and lipid particles [136]. CD36 deficiency impairs macrophage phagocytosis of ACs and alters inflammatory cytokine production following exposure to oxidized lipids [137]. Oxidatively modified lipids are abundant in inflammatory environments; such interactions may influence the persistence of apoptotic debris and immune complexes in lupus tissues [138].

Among the SRs associated with ACs recognition, scavenger receptor class F member 1 (SCARF1) has received particular attention. SCARF1 is expressed on endothelial cells and antigen-presenting cells where it binds ACs through exposed phosphatidylserine and the complement receptor C1q. Genetic deletion of *Scarf1* in mice results in defective clearance of ACs, resulting in the spontaneous development of lupus-like autoimmunity characterized by circulating autoantibodies, immune complex deposition in tissues and tissue damage [30]. Mechanistic analyses demonstrated that SCARF1-mediated uptake of apoptotic cells promotes anti-inflammatory signaling in human DCs, including production of regulatory cytokines that limit inflammatory responses to self-antigens [139]. These findings support a model in which efficient clearance through specific scavenger receptors not only removes immunogenic material but also actively promotes immunological tolerance.

Defects in ACs clearance have downstream consequences for antigen availability and immune activation [140]. When apoptotic debris persists, nucleic acids and associated proteins can remain extracellular or form immune complexes that are subsequently internalized by antigen-presenting cells. This process increases the probability that endogenous nucleic acids reach intracellular compartments capable of triggering innate sensing pathways. In experimental models, delayed clearance of apoptotic material enhances DC activation and promotes the presentation of nuclear antigens to autoreactive lymphocytes [141]. Thus, clearance pathways function as an early regulatory checkpoint that determines whether cellular debris is removed silently or instead becomes a source of inflammatory stimulation.

Human studies provide additional support for impaired clearance mechanisms in lupus. Macrophages isolated from patients with SLE frequently demonstrate reduced phagocytic uptake of ACs in vitro compared with macrophages from healthy donors [142]. Moreover, circulating apoptotic microparticles and nucleosome-containing immune complexes are elevated in patients with active disease, indicating incomplete removal of dying cells in vivo [143]. Although multiple receptor systems contribute to this process, reduced expression or altered function of receptors involved in apoptotic cell recognition may contribute to these defects.

The consequences of defective clearance extend beyond antigen accumulation to include alterations in immune regulation. Under physiological conditions, engulfment of ACs induces anti-inflammatory signaling programs in phagocytes, including production of IL-10 and TGF-β, which promote immune tolerance and suppress inflammatory cytokine production [144]. Failure to efficiently remove apoptotic material therefore disrupts these tolerogenic signals and favors the persistence of inflammatory responses. In the context of lupus, such disturbances can facilitate the activation of autoreactive lymphocytes and perpetuate systemic immune dysregulation.

Environmental and metabolic factors may further influence SRs function. Oxidative stress and lipid peroxidation modify cellular membranes and lipoproteins, generating ligands that interact with scavenger receptors while simultaneously altering phagocytic signaling pathways [145]. Oxidized lipid species can interfere with efficient apoptotic cell uptake and promote inflammatory signaling in macrophages [146,147]. These observations suggest that metabolic stress and inflammatory conditions present in lupus tissues may indirectly exacerbate defects in debris clearance.

Taken together, current evidence indicates that SRs-mediated clearance pathways play an important role in maintaining immune tolerance by removing ACs and limiting the availability of immunogenic self-antigens. Disruption of efferocytosis can promote accumulation of nuclear material, altered phagocyte signaling, and enhanced activation of adaptive immune responses. Rather than acting as isolated pathogenic drivers, SRs operate within a broader network of clearance mechanisms whose collective failure contributes to the persistence of autoantigens and the propagation of systemic autoimmunity in SLE.

## 9. Metabolic and Cellular Reprograming in SLE

SLE is recognized as a disorder characterized by substantial metabolic remodeling within immune and tissue cells [148]. Cellular metabolism not only provides energy for immune activation but also influences differentiation, survival, and effector functions of immune populations [148]. Accumulating evidence indicates that immune cells in lupus undergo metabolic adaptations that support persistent activation and inflammatory signaling [149]. These metabolic alterations arise from chronic immune stimulation, oxidative stress, and altered intracellular signaling pathways that collectively reshape cellular bioenergetics.

T lymphocytes provide some of the clearest examples of metabolic reprograming in lupus. Peripheral T cells from patients with SLE have revealed abnormalities in mitochondrial function, including mitochondrial hyperpolarization, increased production of reactive oxygen species, and altered ATP generation [150]. These mitochondrial perturbations influence downstream signaling pathways and contribute to enhanced T cell activation and survival. Furthermore, increased oxidative stress within lupus T cells also promotes activation of redox-sensitive signaling cascades that regulate cytokine production and cellular differentiation [126].

Metabolic pathway utilization is also altered during T cell activation in lupus. Activated T cells typically increase glycolysis to support rapid proliferation and biosynthesis, a process often referred to as metabolic reprograming [148]. Evidence from both human studies and murine lupus models indicates that lupus T cells display enhanced glycolytic activity and altered mitochondrial metabolism compared with cells from healthy individuals [123]. These metabolic features can influence differentiation of specific T helper subsets, including Th17 and T follicular helper cells, which have been implicated in lupus pathogenesis.

B cells also undergo metabolic adaptations that influence their differentiation and antibody production. During activation and differentiation into plasma cells, B cells increase mitochondrial respiration and biosynthetic pathways required for immunoglobulin synthesis [151]. Experimental studies have demonstrated that plasma cell differentiation is supported by metabolic programs regulated by transcription factors such as BLIMP-1 and XBP1, which coordinate cellular metabolism with antibody production [148]. Due to the proinflammatory environment of lupus, these metabolic programs may contribute to the sustained generation of antibody-secreting cells.

Innate immune cells similarly exhibit metabolic alterations during lupus inflammation. Monocytes and DCs exposed to immune complexes and inflammatory cytokines demonstrate increased glycolysis and altered mitochondrial metabolism, metabolic features associated with proinflammatory activation states [152,153]. These metabolic changes support cytokine production and antigen presentation, thereby amplifying immune responses. Importantly, such metabolic shifts are not unique to lupus but reflect broader principles of immunometabolism observed across inflammatory conditions.

There is a close interaction between metabolism and epigenetic regulation of gene expression. Cellular metabolites such as acetyl-CoA, α-ketoglutarate, and S-adenosylmethionine serve as substrates for chromatin-modifying enzymes that regulate histone acetylation and DNA methylation [154]. Consequently, alterations in metabolic pathways can directly influence chromatin structure and transcriptional programs in immune cells [153]. Hypomethylation of interferon-regulated genes in CD4^+^ T cells, CD19^+^ B cells, and CD14^+^ monocytes has been linked to altered metabolite availability and persistent inflammatory gene activation in SLE [155,156].

During lupus, metabolic disturbances affect immune cells and tissues targeted by inflammatory response. Endothelial cells exposed to inflammatory cytokines and immune complexes demonstrate metabolic shifts toward glycolysis, which supports increased expression of adhesion molecules and recruitment of leukocytes [157]. Similarly, renal cells in lupus nephritis exhibit mitochondrial dysfunction, oxidative stress, and altered lipid metabolism, changes that contribute to tissue injury and fibrosis. These observations highlight the systemic nature of metabolic remodeling in lupus.

Additional studies support the idea that metabolic pathways influence disease progression. In murine lupus models (MRL/lpr and NZB/W F1), pharmacologic modulation of metabolic regulators such as mTOR signaling has been shown to normalize T cell activation and reduce disease severity [126,158,159]. These findings suggest that metabolic interventions may complement immunomodulatory therapies by altering the cellular programs that sustain chronic immune activation.

Overall, metabolic and cellular reprograming represent an important dimension of lupus pathogenesis that links intracellular bioenergetics with immune cell activation and tissue injury. Rather than acting independently, metabolic pathways interact with signaling networks, transcriptional programs, and environmental cues to shape immune responses. Understanding how metabolic alterations influence immune function may therefore provide new opportunities for therapeutic intervention in systemic lupus erythematosus.

## 10. Organ-Specific Pathophysiology

Although systemic immune dysregulation is a defining feature of SLE, the clinical manifestations of the disorder arise from tissue-specific responses to circulating autoantibodies, immune complexes, and inflammatory mediators. Different organs exhibit distinct susceptibilities to immune-mediated injury, reflecting variations in cellular composition, vascular architecture, and intrinsic stress responses. Consequently, the pattern of organ involvement in lupus reflects the interaction between systemic immune abnormalities and local tissue environments.

The kidney is one of the most extensively studied target organs in lupus. Lupus nephritis (LN) develops when circulating immune complexes containing nuclear antigens deposit in glomerular structures, particularly within the mesangium and along the glomerular basement membrane [1,160]. In turn, the antigen deposits activate complement pathways and recruit inflammatory cells, initiating local inflammatory cascades that damage renal tissues [161]. Resident kidney cells, including mesangial cells, podocytes, and tubular epithelial cells, respond to immune complexes and inflammatory cytokines by producing chemokines and proinflammatory mediators that further amplify local inflammation [162]. This results in the most common and severe manifestation of SLE, with up to 60% of SLE patients being affected.

Podocytes are particularly sensitive to inflammatory and oxidative stress in lupus nephritis [163]. Wright et al. showed that exposure to inflammatory cytokines and immune complexes alters cytoskeletal integrity and survival pathways in podocytes, contributing to proteinuria and glomerular dysfunction [164]. In addition, renal macrophages and DCs accumulate within inflamed glomeruli and interstitial compartments, where they participate in cytokine production and antigen presentation that sustain renal inflammation. Immune cells also play a role in the pathogenesis of lupus nephritis. For example, IL-17-producing double-negative T-cells, a group of heterogenous T lymphocytes lacking CD4 and CD8, have shown pathogenic importance by producing IL-17 and IFNγ [165,166]. Furthermore, sustained activation of B-cell receptor by autoantigens leads to chronic inflammation, resulting in higher disease activity [167].

Cardiovascular complications are another major contributor to morbidity in SLE. Patients with SLE exhibit increased risk of accelerated atherosclerosis and vascular dysfunction compared with the general population [168]. Epidemiologic studies indicate that cardiovascular disease is a leading cause of late-stage mortality in SLE, with young women demonstrating up to a 50-fold increased risk of myocardial infarction compared with age-matched controls [169]. Importantly, traditional cardiovascular risk scores underestimate this risk, as SLE-specific factors, including chronic systemic inflammation, immune complex deposition, and autoantibody-mediated endothelial injury, contribute independently to atherogenesis [170]. Endothelial cells exposed to inflammatory cytokines and immune complexes display increased expression of adhesion molecules such as VCAM-1 and ICAM-1, promoting leukocyte recruitment to vascular tissues. Chronic inflammation, oxidative stress, and autoantibody-mediated endothelial injury collectively contribute to vascular damage and thrombosis. Neurological manifestations of lupus illustrate the complex interaction between systemic immune responses and the central nervous system [171]. Neuropsychiatric (NP) SLE encompasses a spectrum of symptoms, including cognitive dysfunction, seizures, and mood disturbances [172]. Entry of circulating autoantibodies and inflammatory mediators into the central nervous system is thought to require disruption of the blood–brain barrier (BBB), which increases its permeability and permits access to neural tissue [173]. Experimental and clinical studies have demonstrated that inflammatory cytokines, complement activation, immune complexes, and endothelial injury in SLE can disrupt BBB integrity by altering tight junction organization and increasing vascular permeability [174]. This increased permeability enables circulating neurotoxic autoantibodies, including anti-NR2 glutamate receptor antibodies and antiphospholipid antibodies, to penetrate brain tissue where they can directly interact with neuronal and endothelial targets [175]. Animal studies showed that anti-NR2 antibodies derived from lupus patients induce neuronal apoptosis only when BBB disruption is experimentally induced, demonstrating that barrier compromise is necessary for pathogenic antibody access to the brain [176,177]. In addition, cytokines such as TNF-α and IL-6, together with type I interferon-mediated endothelial activation, further amplify leukocyte trafficking and local neuroinflammation within the CNS [178]. Imaging and cerebrospinal fluid studies in patients with NPSLE have also provided evidence of increased BBB permeability associated with neurocognitive dysfunction, seizures, mood disorders, and other neuropsychiatric manifestations [179,180].

NPSLE is the least understood complication of the disease; however, it is one of the most prevalent manifestations of lupus [181]. Studies suggest that autoantibodies targeting neuronal antigens, as well as inflammatory cytokines and immune complexes, may alter neuronal signaling and synaptic function [181,182]. These autoantibodies can directly bind to neuronal receptors or ion channels, disrupting synaptic transmission, while inflammatory cytokines such as IL-6 and TNF-α modulate neurotransmitter release and impair neuronal plasticity, collectively leading to cognitive and behavioral deficits. Activation of microglia and astrocytes within the central nervous system can further amplify inflammatory responses and contribute to neuronal injury [183]. Sustained neuroinflammation can also promote excitotoxicity and oxidative stress, exacerbating neuronal damage and functional impairment in NPSLE.

The skin represents another organ commonly affected in lupus. Research has shown that the skin serves as an interface between environmental exposures and immune responses [184]. Cutaneous lupus erythematosus (CLE) manifests through immune-mediated lesions driven by both genetic susceptibility and environmental triggers. Several immune genes have been implicated in subtypes of CLE, including cytokine genes, complement genes, and innate immune genes [185,186]. Polymorphisms in immune-related genes, including IRF5, TYK2, and CTLA4, have been associated with CLE subtypes, while rare forms such as familial chilblain lupus result from gain-of-function mutations in STING [187,188] or loss-of-function mutations in TREX1 [185,189,190,191]. UV radiation can induce apoptosis in keratinocytes and promote the release of nuclear antigens and inflammatory mediators. These factors activate resident immune cells and promote recruitment of pDCs and lymphocytes, leading to the inflammatory lesions characteristic of cutaneous lupus [48]. The skin therefore illustrates how environmental triggers interact with systemic immune dysregulation to produce localized pathology. CLE represents a spectrum of disease that may occur either as a skin-restricted condition or as part of SLE [192]; however, CLE may occur in the absence of clinically evident systemic disease while it shares core immunopathogenic pathways with SLE [193]. Despite their distinct clinical presentations, CLE and SLE share core immunopathogenic mechanisms, including type I interferon-driven inflammation [8], nucleic acid sensing pathways [194,195] and overlapping genetic susceptibility loci [196]. In CLE, these systemic predispositions are strongly shaped by local environmental factors, particularly ultraviolet radiation, which amplifies keratinocyte injury and antigen release, leading to compartmentalized cutaneous inflammation [196]. In contrast, SLE reflects a systemic failure of immune tolerance affecting multiple organ systems [1]. Accordingly, CLE can be viewed as a tissue-restricted manifestation within the broader immunological continuum of SLE, in which local environmental cues determine organ-specific expression of shared immune dysregulation [197].

Over the past decade, the gut has emerged as a relevant site in the development and progression of SLE [38,198,199,200]. The intestine represents a major immunological interface, where continuous interactions between host immunity and commensal microbes contribute to inflammatory balance, metabolic regulation, and immune tolerance [201,202]. Experimental evidence demonstrates that alterations in the gut microbiota (dysbiosis) can directly influence systemic autoimmunity [203,204]. In lupus-prone mouse models (e.g., NZB/W F1 and MRL/lpr), transplantation of gut microbiota from diseased mice into germ-free or antibiotic-treated mice accelerates autoantibody production and renal pathology, demonstrating causality [203,205]. Mechanistically, dysbiosis promotes increased intestinal permeability, allowing microbial components such as lipopolysaccharide (LPS) to translocate into circulation, triggering systemic immune activation [204]. Short-chain fatty acids produced by commensals regulate regulatory T cell differentiation and inflammatory cytokine production; their reduction in lupus models correlates with heightened Th17/Treg imbalance [205]. Human studies corroborate these findings; SLE patients show reproducible decreases in Firmicutes and increases in Bacteroidetes, along with elevated markers of microbial translocation, which correlate with disease activity [199,204].

In SLE, defects in apoptotic cell clearance (efferocytosis) contribute to the accumulation of secondary necrotic material and sustained release of inflammatory mediators. These processes may extend to mucosal tissues, where they can disrupt epithelial integrity and alter host–microbe interactions. Impaired barrier function, often described as increased intestinal permeability, provides a route for microbial products to access systemic circulation, thereby linking local barrier dysfunction with systemic immune activation [200].

Consistent with this model, studies in SLE and other autoimmune diseases have reported changes in gut microbiome composition alongside evidence of barrier dysfunction. However, most human studies in lupus remain largely descriptive, and the extent to which microbiome alterations represent primary drivers versus downstream consequences of immune dysregulation remains unresolved [198,200]. Mechanistic insights into how defects in immune clearance pathways shape the gut microbial landscape and contribute to systemic inflammation are still limited. The gut microbiome can be viewed as a dynamic and potentially modifiable component of lupus pathogenesis. Shifts in microbial communities may influence epithelial integrity, metabolite production, and immune signaling, thereby reinforcing inflammatory pathways. While current evidence supports a bidirectional relationship between systemic autoimmunity and gut homeostasis, further studies are required to establish causality and define how these interactions may be therapeutically targeted [198,200].

Additional organs, including the lungs, liver, and hematopoietic system, may also be affected in lupus through mechanisms involving immune complex deposition, cytokine-mediated inflammation, and autoantibody-mediated cell injury. The specific pattern of organ involvement varies among patients, reflecting differences in genetic susceptibility, immune responses, and environmental exposures. Collectively, organ-specific pathology in lupus emerges from the interplay between systemic autoimmunity and local tissue responses. Circulating immune complexes, inflammatory cytokines, and autoreactive immune cells interact with organ-specific cell populations to produce tissue injury. These insights set the stage for therapeutic approaches that are not only systemic but also tailored to the vulnerabilities of specific organs, bridging molecular pathogenesis with clinical management.

## 11. Translational Implications and Therapeutic Strategies

Advances in understanding SLE pathogenesis, from genetic susceptibility and epigenetic regulation to immune dysregulation within affected organs, have reshaped strategies for therapeutic development. However, the heterogeneity of clinical phenotypes and fluctuating interferon signatures caution against uniform therapeutic assumptions. Effective intervention requires selective modulation of pathogenic circuits while preserving antimicrobial competence, particularly given the infection-related morbidity associated with global immunosuppression. Integration of molecular endotypes with clinical phenotypes, including interferon-high versus interferon-low subsets, increasingly guides rational trial design and therapeutic selection [8,69].

Targeting nucleic acid sensing and type I IFN pathways represents one of the most substantiated mechanism-based approaches in SLE. Elevated interferon-stimulated gene expression in peripheral blood and affected tissues correlates with disease activity and renal involvement [206,207]. Therapeutic neutralization of IFN-α with sifalimumab demonstrated suppression of interferon gene signatures and improvement in cutaneous and musculoskeletal endpoints in phase II studies [208]. More definitively, the anti-IFNAR1 monoclonal antibody anifrolumab achieved clinical efficacy in moderate to severe SLE in the TULIP trials, particularly in patients with high baseline interferon signatures [86,87,209]. These data validate IFN receptor blockade as a disease-modifying strategy, although incomplete responses underscore parallel pathogenic axes. Inhibition of upstream sensors such as TLR7/9 has shown disease attenuation in murine lupus [210], and early-phase human studies with TLR antagonists have demonstrated biological activity, though sustained clinical efficacy remains under evaluation. Similarly, pharmacologic modulation of the cGAS-STING pathway shows promise in preclinical models, but translation requires careful titration given its role in antiviral defense.

B-cell-directed therapies remain central, yet clinical outcomes highlight complexity beyond simple depletion paradigms. Rituximab, despite mechanistic rationale, did not meet primary endpoints in randomized SLE trials, likely reflecting trial design and background immunosuppression rather than absence of biologic effect [211]. In contrast, belimumab, a monoclonal antibody targeting BAFF, demonstrated modest but reproducible efficacy and remains an approved therapy for systemic and renal SLE [100,209]. These findings emphasize that selective modulation of B cell survival signals may preferentially affect autoreactive subsets while preserving protective immunity. Dual BAFF/APRIL blockade with atacicept showed biologic activity but raised concerns regarding infection risk and hypogammaglobulinemia [212], illustrating the balance required between efficacy and immune competence. Combination strategies integrating B cell modulation with interferon blockade are being explored to address interconnected adaptive and innate circuits.

T-cell-targeted interventions are supported by evidence linking Tfh expansion and Treg instability to disease activity [113]. Abatacept, a CTLA4-Ig fusion protein inhibiting CD28-mediated co-stimulation, demonstrated mixed results in lupus nephritis trials, though mechanistic endpoints suggested biologic engagement [213]. Blockade of CD40–CD40L interactions, previously limited by thromboembolic complications, is being revisited with safer antibody engineering approaches. Low-dose interleukin-2 therapy has emerged as a strategy to selectively expand regulatory T cells and restore immune tolerance, showing promising phase I/II outcomes with improved disease activity indices [214]. These data support calibrated immune rebalancing rather than broad T cell suppression.

Restoration of defective clearance pathways represents a biologically coherent yet clinically underdeveloped avenue. Impaired efferocytosis contributes to persistent autoantigen exposure and interferon amplification. Experimental augmentation of phagocytic receptors such as SCARF1 enhances ACs clearance and reduces lupus-like pathology in murine systems [30]. Complement replacement or modulation strategies are similarly under investigation, particularly in lupus nephritis, where complement activation contributes to tissue injury. Targeted inhibition of C5 with eculizumab has shown benefit in refractory complement-mediated manifestations, though controlled lupus-specific trials remain limited.

Immunometabolic modulation has gained translational traction following demonstration of heightened glycolysis and mitochondrial hyperpolarization in lupus T cells [123]. In lupus-prone mice, combined inhibition of glycolysis and mitochondrial metabolism normalized T cell activation and reduced autoantibody production [123]. mTOR inhibition with rapamycin improved disease activity and normalized T cell subsets in small clinical studies [125,215], supporting metabolic recalibration as a therapeutic principle. These findings position immunometabolism as a convergence point linking interferon signaling, lymphocyte activation, and organ damage.

Organ-directed therapy has progressed most substantially in LN. The addition of voclosporin, a calcineurin inhibitor with improved pharmacokinetics, to standard therapy increased renal response rates in the AURORA trial [215]. Belimumab also demonstrated additive benefit in renal SLE [87]. Complement pathway inhibitors and anti-inflammatory biologics targeting intrarenal cytokine networks are in development, reflecting recognition that systemic immune modulation must be complemented by tissue-specific protection. Neuropsychiatric manifestations require strategies that account for blood–brain barrier disruption, enabling immune factor entry and localized CNS immune activation. The integration of multi-omics approaches, including genomics, transcriptomics, epigenomics, and single-cell profiling, provides predictive biomarkers for patient stratification and therapeutic response and may guide adaptive treatment algorithms [69]. Personalized medicine strategies can leverage these biomarkers to identify patients who are most likely to benefit from targeted interventions, monitor disease trajectory, and adjust therapy dynamically.

Collectively, translational strategies in SLE increasingly move beyond broad immunosuppression toward a multi-pronged, mechanism-based approach. Combining systemic and organ-specific therapies, modulating innate and adaptive immunity, correcting metabolic and clearance defects, and integrating predictive biomarkers offers a roadmap for durable disease control and improved quality of life. Mechanistic insights from murine and human studies, including previous studies from our lab provide both rationale and proof-of-concept for these emerging interventions, demonstrating that precision immunology can transform SLE management.

## 12. Concluding Perspective

Systemic lupus erythematosus exemplifies how convergent perturbations in innate sensing, adaptive immunity, clearance pathways, and metabolic regulation generate systemic yet organ-selective pathology. Rather than a single dominant defect, SLE reflects layered dysregulation in which genetic susceptibility, environmental exposures, and stochastic immune activation intersect to sustain autoreactivity. The cumulative experimental and clinical literature demonstrates that persistent nucleic acid-driven interferon signaling, aberrant B and T cell interactions, and defective resolution of apoptotic material form an interconnected pathogenic scaffold [1,8].

Therapeutic evolution over the past decade substantiates the principle that precise pathway targeting can modify disease trajectory. Interferon receptor blockade, BAFF inhibition, metabolic modulation, and selective co-stimulatory interference each provide partial but reproducible benefit, reinforcing the multifactorial nature of disease maintenance. Importantly, clinical variability underscores that no single axis fully accounts for pathology across all patients, necessitating stratified and potentially combinatorial regimens.

Advances in multiomic integration further redefine SLE as a spectrum of molecular endotypes rather than a uniform entity. High-dimensional immune profiling enables correlation of cellular states with organ involvement and therapeutic response, fostering predictive modeling that anticipates flare risk and treatment failure. Although validation in large, diverse cohorts remains ongoing, this framework supports a transition from reactive flare suppression to proactive immune recalibration.

Future progress depends on harmonizing systemic immune modulation with organ-protective strategies and dynamic biomarker monitoring. Durable remission will likely require coordinated targeting of innate sensing, adaptive activation, and tissue-specific inflammatory circuits while safeguarding host defense. Continued integration of mechanistic insight with rigorously designed clinical trials offers the clearest path toward sustained disease control.

In this context, SLE serves not only as a clinical challenge but also as a paradigm for complex autoimmune disease. The iterative dialogue between mechanistic discovery and therapeutic innovation demonstrates that precision immunology can translate into tangible clinical benefit when guided by robust experimental validation and careful clinical stratification.

## Figures and Tables

**Table 1:** Genetic architecture of systemic lupus erythematosus.

Gene	Representative Variant (SNP)	Functional Category	Proposed Immunological Effect	Key Reference
*irf5*	rs2004640, rs10954213	Transcription factor regulating interferon responses	Increased IRF5 expression and enhanced type I interferon and pro-inflammatory cytokine transcription	Graham RR et al. [12]
*stat4*	rs7574865	Cytokine signaling transcription factor	Increased STAT4 signaling downstream of IL-12 and type I IFN, promoting Th1 differentiation and inflammatory responses	Remmers EF et al. [24]
*blk*	rs13277113	B-cell receptor signaling kinase	Altered BCR signaling threshold affecting B-cell activation and tolerance checkpoints	Hom G et al. [11]
*bank1*	rs10516487	B-cell adaptor protein	Modifies calcium signaling and BCR pathway activation, influencing autoreactive B-cell survival	Kozyrev SV et al. [25]
*Itam* (CD11b)	rs1143679	Complement receptor/phagocytosis receptor	Impaired clearance of immune complexes and apoptotic debris	Rhodes et al. [26]
HLA-DRB1/HLA-DQ	Multiple alleles	Antigen presentation (MHC class II)	Alters peptide presentation and autoreactive T-cell activation	Tsokos GC et al. [1]
Complement C4A copy number	CNV (low copy number)	Complement pathway	Reduced immune-complex and apoptotic debris clearance	Yang Y et al. [27]
*tnfaip3* (A20)	rs2230926	Negative regulator of NF-κB signaling	Reduced A20 inhibitory activity leading to prolonged inflammatory signaling	Graham RR et al. [12]
*ptpn22*	rs2476601	Protein tyrosine phosphatase regulating antigen receptor signaling	Alters lymphocyte activation thresholds and immune tolerance	Gateva V et al. [23]

## Data Availability

No new datasets were generated for this review. Any supporting information or supplementary material is available upon reasonable request from the corresponding author.

## References

[1] Tsokos G.C. (2011). Systemic lupus erythematosus. N. Engl. J. Med..

[2] Kaul A., Gordon C., Crow M.K., Touma Z., Urowitz M.B., van Vollenhoven R., Ruiz-Irastorza G., Hughes G. (2016). Systemic lupus erythematosus. Nat. Rev. Dis. Prim..

[3] Lech M., Anders H.J. (2013). The pathogenesis of lupus nephritis. J. Am. Soc. Nephrol..

[4] Bentham J., Morris D.L., Graham D.S.C., Pinder C.L., Tombleson P., Behrens T.W., Martin J., Fairfax B.P., Knight J.C., Chen L. (2015). Genetic association analyses implicate aberrant regulation of innate and adaptive immunity genes in the pathogenesis of systemic lupus erythematosus. Nat. Genet..

[5] Morris D.L., Sheng Y., Zhang Y., Wang Y.F., Zhu Z., Tombleson P., Chen L., Cunninghame Graham D.S., Bentham J., Roberts A.L. (2016). Genome-wide association meta-analysis in Chinese and European individuals identifies ten new loci associated with systemic lupus erythematosus. Nat. Genet..

[6] Zhang Y., Day K., Absher D.M. (2022). STAT3-mediated allelic imbalance of novel genetic variant Rs1047643 and B-cell-specific super-enhancer in association with systemic lupus erythematosus. eLife.

[7] Lu X., Chen X., Forney C., Donmez O., Miller D., Parameswaran S., Hong T., Huang Y., Pujato M., Cazares T. (2021). Global discovery of lupus genetic risk variant allelic enhancer activity. Nat. Commun..

[8] Crow M.K. (2014). Type I interferon in the pathogenesis of lupus. J. Immunol..

[9] Arazi A., Rao D.A., Berthier C.C., Davidson A., Liu Y., Hoover P.J., Chicoine A., Eisenhaure T.M., Jonsson A.H., Li S. (2019). The immune cell landscape in kidneys of patients with lupus nephritis. Nat. Immunol..

[10] Lin S.Y., Yu Y., Nie D., Yang L., Chen Y., Chen Y., Wen C., Zeng Z. (2025). Single-cell RNA sequencing reveals the heterogeneity and regulatory modules of cell-specific RNA-binding proteins in patients with systemic lupus erythematosus. Biochem. Biophys. Rep..

[11] Harley J.B., Alarcon-Riquelme M.E., Criswell L.A., Jacob C.O., Kimberly R.P., Moser K.L., Tsao B.P., Vyse T.J., Langefeld C.D., The International Consortium for Systemic Lupus Erythematosus Genetics (SLEGEN) (2008). Genome-wide association scan in women with systemic lupus erythematosus identifies susceptibility variants in *ITGAM*, *PXK*, *KIAA1542* and other loci. Nat. Genet..

[12] Graham R.R., Cotsapas C., Davies L., Hackett R., Lessard C.J., Leon J.M., Burtt N.P., Guiducci C., Parkin M., Gates C. (2008). Genetic variants near TNFAIP3 on 6q23 are associated with systemic lupus erythematosus. Nat. Genet..

[13] Yin X., Kim K., Suetsugu H., Bang S.Y., Wen L., Koido M., Ha E., Liu L., Sakamoto Y., Jo S. (2021). Meta-analysis of 208370 East Asians identifies 113 susceptibility loci for systemic lupus erythematosus. Ann. Rheum. Dis..

[14] Farh K.K., Marson A., Zhu J., Kleinewietfeld M., Housley W.J., Beik S., Shoresh N., Whitton H., Ryan R.J., Shishkin A.A. (2015). Genetic and epigenetic fine mapping of causal autoimmune disease variants. Nature.

[15] Deplancke B., Alpern D., Gardeux V. (2016). The Genetics of Transcription Factor DNA Binding Variation. Cell.

[16] Roadmap Epigenomics C., Kundaje A., Meuleman W., Ernst J., Bilenky M., Yen A., Heravi-Moussavi A., Kheradpour P., Zhang Z., Wang J. (2015). Integrative analysis of 111 reference human epigenomes. Nature.

[17] Trynka G., Sandor C., Han B., Xu H., Stranger B.E., Liu X.S., Raychaudhuri S. (2013). Chromatin marks identify critical cell types for fine mapping complex trait variants. Nat. Genet..

[18] Raj T., Rothamel K., Mostafavi S., Ye C., Lee M.N., Replogle J.M., Feng T., Lee M., Asinovski N., Frohlich I. (2014). Polarization of the effects of autoimmune and neurodegenerative risk alleles in leukocytes. Science.

[19] Fairfax B.P., Humburg P., Makino S., Naranbhai V., Wong D., Lau E., Jostins L., Plant K., Andrews R., McGee C. (2014). Innate immune activity conditions the effect of regulatory variants upon monocyte gene expression. Science.

[20] Graham R.R., Kyogoku C., Sigurdsson S., Vlasova I.A., Davies L.R., Baechler E.C., Plenge R.M., Koeuth T., Ortmann W.A., Hom G. (2007). Three functional variants of IFN regulatory factor 5 (IRF5) define risk and protective haplotypes for human lupus. Proc. Natl. Acad. Sci. USA.

[21] Cham C.M., Ko K., Niewold T.B. (2012). Interferon regulatory factor 5 in the pathogenesis of systemic lupus erythematosus. Clin. Dev. Immunol..

[22] Musone S.L., Taylor K.E., Lu T.T., Nititham J., Ferreira R.C., Ortmann W., Shifrin N., Petri M.A., Kamboh M.I., Manzi S. (2008). Multiple polymorphisms in the TNFAIP3 region are independently associated with systemic lupus erythematosus. Nat. Genet..

[23] Gateva V., Sandling J.K., Hom G., Taylor K.E., Chung S.A., Sun X., Ortmann W., Kosoy R., Ferreira R.C., Nordmark G. (2009). A large-scale replication study identifies TNIP1, PRDM1, JAZF1, UHRF1BP1 and IL10 as risk loci for systemic lupus erythematosus. Nat. Genet..

[24] Rovin B.H., Teng Y.K.O., Ginzler E.M., Arriens C., Caster D.J., Romero-Diaz J., Gibson K., Kaplan J., Lisk L., Navarra S. (2021). Efficacy and safety of voclosporin versus placebo for lupus nephritis (AURORA 1): A double-blind, randomised, multicentre, placebo-controlled, phase 3 trial. Lancet.

[25] Remmers E.F., Plenge R.M., Lee A.T., Graham R.R., Hom G., Behrens T.W., de Bakker P.I., Le J.M., Lee H.S., Batliwalla F. (2007). STAT4 and the risk of rheumatoid arthritis and systemic lupus erythematosus. N. Engl. J. Med..

[26] Kozyrev S.V., Abelson A.K., Wojcik J., Zaghlool A., Linga Reddy M.V., Sanchez E., Gunnarsson I., Svenungsson E., Sturfelt G., Jonsen A. (2008). Functional variants in the B-cell gene BANK1 are associated with systemic lupus erythematosus. Nat. Genet..

[27] Yang Y., Chung E.K., Wu Y.L., Savelli S.L., Nagaraja H.N., Zhou B., Hebert M., Jones K.N., Shu Y., Kitzmiller K. (2007). Gene copy-number variation and associated polymorphisms of complement component C4 in human systemic lupus erythematosus (SLE): Low copy number is a risk factor for and high copy number is a protective factor against SLE susceptibility in European Americans. Am. J. Hum. Genet..

[28] Munoz L.E., Lauber K., Schiller M., Manfredi A.A., Herrmann M. (2010). The role of defective clearance of apoptotic cells in systemic autoimmunity. Nat. Rev. Rheumatol..

[29] Nagata S., Segawa K. (2021). Sensing and clearance of apoptotic cells. Curr. Opin. Immunol..

[30] Ramirez-Ortiz Z.G., Pendergraft W.F., Prasad A., Byrne M.H., Iram T., Blanchette C.J., Luster A.D., Hacohen N., El Khoury J., Means T.K. (2013). The scavenger receptor SCARF1 mediates the clearance of apoptotic cells and prevents autoimmunity. Nat. Immunol..

[31] Tsokos G.C., Lo M.S., Costa Reis P., Sullivan K.E. (2016). New insights into the immunopathogenesis of systemic lupus erythematosus. Nat. Rev. Rheumatol..

[32] Hedrich C.M. (2017). Epigenetics in SLE. Curr. Rheumatol. Rep..

[33] Cornacchia E., Golbus J., Maybaum J., Strahler J., Hanash S., Richardson B. (1988). Hydralazine and procainamide inhibit T cell DNA methylation and induce autoreactivity. J. Immunol..

[34] Adams D.E., Shao W.H. (2022). Epigenetic Alterations in Immune Cells of Systemic Lupus Erythematosus and Therapeutic Implications. Cells.

[35] Richardson B. (2003). DNA methylation and autoimmune disease. Clin. Immunol..

[36] Kuhn N.F., Purdon T.J., van Leeuwen D.G., Lopez A.V., Curran K.J., Daniyan A.F., Brentjens R.J. (2019). CD40 Ligand-Modified Chimeric Antigen Receptor T Cells Enhance Antitumor Function by Eliciting an Endogenous Antitumor Response. Cancer Cell.

[37] Javierre B.M., Fernandez A.F., Richter J., Al-Shahrour F., Martin-Subero J.I., Rodriguez-Ubreva J., Berdasco M., Fraga M.F., O’Hanlon T.P., Rider L.G. (2010). Changes in the pattern of DNA methylation associate with twin discordance in systemic lupus erythematosus. Genome Res..

[38] Mu S., Wang W., Liu Q., Ke N., Li H., Sun F., Zhang J., Zhu Z. (2024). Autoimmune disease: A view of epigenetics and therapeutic targeting. Front. Immunol..

[39] Djuranovic S., Nahvi A., Green R. (2012). miRNA-mediated gene silencing by translational repression followed by mRNA deadenylation and decay. Science.

[40] Pan W., Zhu S., Yuan M., Cui H., Wang L., Luo X., Li J., Zhou H., Tang Y., Shen N. (2010). MicroRNA-21 and microRNA-148a contribute to DNA hypomethylation in lupus CD4+ T cells by directly and indirectly targeting DNA methyltransferase 1. J. Immunol..

[41] Qu B., Shen N. (2015). miRNAs in the Pathogenesis of Systemic Lupus Erythematosus. Int. J. Mol. Sci..

[42] Tang Y., Luo X., Cui H., Ni X., Yuan M., Guo Y., Huang X., Zhou H., de Vries N., Tak P.P. (2009). MicroRNA-146A contributes to abnormal activation of the type I interferon pathway in human lupus by targeting the key signaling proteins. Arthritis Rheum..

[43] Pauley K.M., Cha S., Chan E.K. (2009). MicroRNA in autoimmunity and autoimmune diseases. J. Autoimmun..

[44] Rahman A., Isenberg D.A. (2008). Systemic lupus erythematosus. N. Engl. J. Med..

[45] Cooper G.S., Wither J., Bernatsky S., Claudio J.O., Clarke A., Rioux J.D., Ca N.G.I., Fortin P.R. (2010). Occupational and environmental exposures and risk of systemic lupus erythematosus: Silica, sunlight, solvents. Rheumatology.

[46] Casciola-Rosen L., Rosen A. (1997). Ultraviolet light-induced keratinocyte apoptosis: A potential mechanism for the induction of skin lesions and autoantibody production in LE. Lupus.

[47] Saegusa J., Kawano S., Koshiba M., Hayashi N., Kosaka H., Funasaka Y., Kumagai S. (2002). Oxidative stress mediates cell surface expression of SS-A/Ro antigen on keratinocytes. Free Radic. Biol. Med..

[48] Werth V.P. (2005). Clinical manifestations of cutaneous lupus erythematosus. Autoimmun. Rev..

[49] Kuhn A., Wenzel J., Weyd H. (2014). Photosensitivity, apoptosis, and cytokines in the pathogenesis of lupus erythematosus: A critical review. Clin. Rev. Allergy Immunol..

[50] Furukawa F. (2003). Photosensitivity in cutaneous lupus erythematosus: Lessons from mice and men. J. Dermatol. Sci..

[51] Harley J.B., Chen X., Pujato M., Miller D., Maddox A., Forney C., Magnusen A.F., Lynch A., Chetal K., Yukawa M. (2018). Transcription factors operate across disease loci, with EBNA2 implicated in autoimmunity. Nat. Genet..

[52] James J.A., Kaufman K.M., Farris A.D., Taylor-Albert E., Lehman T.J., Harley J.B. (1997). An increased prevalence of Epstein-Barr virus infection in young patients suggests a possible etiology for systemic lupus erythematosus. J. Clin. Investig..

[53] Munroe M.E., Anderson J.R., Gross T.F., Stunz L.L., Bishop G.A., James J.A. (2020). Epstein-Barr Functional Mimicry: Pathogenicity of Oncogenic Latent Membrane Protein-1 in Systemic Lupus Erythematosus and Autoimmunity. Front. Immunol..

[54] Poole B.D., Gross T., Maier S., Harley J.B., James J.A. (2008). Lupus-like autoantibody development in rabbits and mice after immunization with EBNA-1 fragments. J. Autoimmun..

[55] McClain M.T., Heinlen L.D., Dennis G.J., Roebuck J., Harley J.B., James J.A. (2005). Early events in lupus humoral autoimmunity suggest initiation through molecular mimicry. Nat. Med..

[56] Kamen D.L., Tangpricha V. (2010). Vitamin D and molecular actions on the immune system: Modulation of innate and autoimmunity. J. Mol. Med..

[57] Moulton V.R. (2018). Sex Hormones in Acquired Immunity and Autoimmune Disease. Front. Immunol..

[58] Cutolo M., Sulli A., Capellino S., Villaggio B., Montagna P., Seriolo B., Straub R.H. (2004). Sex hormones influence on the immune system: Basic and clinical aspects in autoimmunity. Lupus.

[59] Bynoe M.S., Grimaldi C.M., Diamond B. (2000). Estrogen up-regulates Bcl-2 and blocks tolerance induction of naive B cells. Proc. Natl. Acad. Sci. USA.

[60] Grimaldi C.M., Cleary J., Dagtas A.S., Moussai D., Diamond B. (2002). Estrogen alters thresholds for B cell apoptosis and activation. J. Clin. Investig..

[61] Shevchuk S., Marynych L., Malovana T., Denyshchych L. (2023). Vitamin D level in patients with systemic lupus erythematosus: Its relationship to disease course and bone mineral density. Lupus Sci. Med..

[62] Almada-Correia I. (2022). Let’s review the gut microbiota in systemic lupus erythematosus. Explor. Med..

[63] Chen Y.F., Hsieh A.H., Wang L.C., Huang Y.J., Yun-Chen T., Tseng W.Y., Kuo Y.L., Luo S.F., Yu K.H., Kuo C.F. (2021). Fecal microbiota changes in NZB/W F1 mice after induction of lupus disease. Sci. Rep..

[64] Luo X.M., Edwards M.R., Mu Q., Yu Y., Vieson M.D., Reilly C.M., Ahmed S.A., Bankole A.A. (2018). Gut Microbiota in Human Systemic Lupus Erythematosus and a Mouse Model of Lupus. Appl. Environ. Microbiol..

[65] Wolf S.J., Estadt S.N., Gudjonsson J.E., Kahlenberg J.M. (2018). Human and Murine Evidence for Mechanisms Driving Autoimmune Photosensitivity. Front. Immunol..

[66] Gonzalez-Quintial R., Nguyen A., Kono D.H., Oldstone M.B.A., Theofilopoulos A.N., Baccala R. (2018). Lupus acceleration by a MAVS-activating RNA virus requires endosomal TLR signaling and host genetic predisposition. PLoS ONE.

[67] Ronnblom L., Pascual V. (2008). The innate immune system in SLE: Type I interferons and dendritic cells. Lupus.

[68] Ronnblom L., Alm G.V. (2003). Systemic lupus erythematosus and the type I interferon system. Arthritis Res. Ther..

[69] Barrat F.J., Elkon K.B., Fitzgerald K.A. (2016). Importance of Nucleic Acid Recognition in Inflammation and Autoimmunity. Annu. Rev. Med..

[70] Kim W.U., Sreih A., Bucala R. (2009). Toll-like receptors in systemic lupus erythematosus; prospects for therapeutic intervention. Autoimmun. Rev..

[71] Eloranta M.L., Alm G.V., Ronnblom L. (2013). Disease mechanisms in rheumatology--tools and pathways: Plasmacytoid dendritic cells and their role in autoimmune rheumatic diseases. Arthritis Rheum..

[72] Shrivastav M., Niewold T.B. (2013). Nucleic Acid sensors and type I interferon production in systemic lupus erythematosus. Front. Immunol..

[73] Liu Y., Pu F. (2023). Updated roles of cGAS-STING signaling in autoimmune diseases. Front. Immunol..

[74] Decout A., Katz J.D., Venkatraman S., Ablasser A. (2021). The cGAS-STING pathway as a therapeutic target in inflammatory diseases. Nat. Rev. Immunol..

[75] Ablasser A., Chen Z.J. (2019). cGAS in action: Expanding roles in immunity and inflammation. Science.

[76] Raz N., Torres I.J., Acker J.D. (1992). Age-related shrinkage of the mamillary bodies: In vivo MRI evidence. Neuroreport.

[77] Seth R.B., Sun L., Ea C.K., Chen Z.J. (2005). Identification and characterization of MAVS, a mitochondrial antiviral signaling protein that activates NF-kappaB and IRF 3. Cell.

[78] Kato H., Takeuchi O., Sato S., Yoneyama M., Yamamoto M., Matsui K., Uematsu S., Jung A., Kawai T., Ishii K.J. (2006). Differential roles of MDA5 and RIG-I helicases in the recognition of RNA viruses. Nature.

[79] Kawai T., Akira S. (2006). Innate immune recognition of viral infection. Nat. Immunol..

[80] Takeuchi O., Akira S. (2010). Pattern recognition receptors and inflammation. Cell.

[81] Gessani S., Conti L., Del Corno M., Belardelli F. (2014). Type I interferons as regulators of human antigen presenting cell functions. Toxins.

[82] Kuka M., De Giovanni M., Iannacone M. (2019). The role of type I interferons in CD4(+) T cell differentiation. Immunol. Lett..

[83] Chen Y., Sharma S., Assis P.A., Jiang Z., Elling R., Olive A.J., Hang S., Bernier J., Huh J.R., Sassetti C.M. (2018). CNBP controls IL-12 gene transcription and Th1 immunity. J. Exp. Med..

[84] Leadbetter E.A., Rifkin I.R., Hohlbaum A.M., Beaudette B.C., Shlomchik M.J., Marshak-Rothstein A. (2002). Chromatin-IgG complexes activate B cells by dual engagement of IgM and Toll-like receptors. Nature.

[85] Scott R.S., McMahon E.J., Pop S.M., Reap E.A., Caricchio R., Cohen P.L., Earp H.S., Matsushima G.K. (2001). Phagocytosis and clearance of apoptotic cells is mediated by MER. Nature.

[86] Furie R., Khamashta M., Merrill J.T., Werth V.P., Kalunian K., Brohawn P., Illei G.G., Drappa J., Wang L., Yoo S. (2017). Anifrolumab, an Anti-Interferon-alpha Receptor Monoclonal Antibody, in Moderate-to-Severe Systemic Lupus Erythematosus. Arthritis Rheumatol..

[87] Furie R., Rovin B.H., Houssiau F., Malvar A., Teng Y.K.O., Contreras G., Amoura Z., Yu X., Mok C.C., Santiago M.B. (2020). Two-Year, Randomized, Controlled Trial of Belimumab in Lupus Nephritis. N. Engl. J. Med..

[88] Dorner T., Lipsky P.E. (2014). B cells: Depletion or functional modulation in rheumatic diseases. Curr. Opin. Rheumatol..

[89] Yurasov S., Wardemann H., Hammersen J., Tsuiji M., Meffre E., Pascual V., Nussenzweig M.C. (2005). Defective B cell tolerance checkpoints in systemic lupus erythematosus. J. Exp. Med..

[90] Dema B., Charles N. (2016). Autoantibodies in SLE: Specificities, Isotypes and Receptors. Antibodies.

[91] Dorner T., Lipsky P.E. (2024). The essential roles of memory B cells in the pathogenesis of systemic lupus erythematosus. Nat. Rev. Rheumatol..

[92] Avalos A.M., Busconi L., Marshak-Rothstein A. (2010). Regulation of autoreactive B cell responses to endogenous TLR ligands. Autoimmunity.

[93] Lau C.M., Broughton C., Tabor A.S., Akira S., Flavell R.A., Mamula M.J., Christensen S.R., Shlomchik M.J., Viglianti G.A., Rifkin I.R. (2005). RNA-associated autoantigens activate B cells by combined B cell antigen receptor/Toll-like receptor 7 engagement. J. Exp. Med..

[94] Shapiro-Shelef M., Calame K. (2005). Regulation of plasma-cell development. Nat. Rev. Immunol..

[95] Su X., Yu H., Lei Q., Chen X., Tong Y., Zhang Z., Yang W., Guo Y., Lin L. (2024). Systemic lupus erythematosus: Pathogenesis and targeted therapy. Mol. Biomed..

[96] Clynes R., Dumitru C., Ravetch J.V. (1998). Uncoupling of immune complex formation and kidney damage in autoimmune glomerulonephritis. Science.

[97] Walport M.J. (2002). Complement and systemic lupus erythematosus. Arthritis Res..

[98] Barr T.A., Shen P., Brown S., Lampropoulou V., Roch T., Lawrie S., Fan B., O’Connor R.A., Anderton S.M., Bar-Or A. (2012). B cell depletion therapy ameliorates autoimmune disease through ablation of IL-6-producing B cells. J. Exp. Med..

[99] Stohl W., Hiepe F., Latinis K.M., Thomas M., Scheinberg M.A., Clarke A., Aranow C., Wellborne F.R., Abud-Mendoza C., Hough D.R. (2012). Belimumab reduces autoantibodies, normalizes low complement levels, and reduces select B cell populations in patients with systemic lupus erythematosus. Arthritis Rheum..

[100] Navarra S.V., Guzman R.M., Gallacher A.E., Hall S., Levy R.A., Jimenez R.E., Li E.K., Thomas M., Kim H.Y., Leon M.G. (2011). Efficacy and safety of belimumab in patients with active systemic lupus erythematosus: A randomised, placebo-controlled, phase 3 trial. Lancet.

[101] Li H., Boulougoura A., Endo Y., Tsokos G.C. (2022). Abnormalities of T cells in systemic lupus erythematosus: New insights in pathogenesis and therapeutic strategies. J. Autoimmun..

[102] Crispin J.C., Kyttaris V.C., Terhorst C., Tsokos G.C. (2010). T cells as therapeutic targets in SLE. Nat. Rev. Rheumatol..

[103] Liossis S.N., Ding X.Z., Dennis G.J., Tsokos G.C. (1998). Altered pattern of TCR/CD3-mediated protein-tyrosyl phosphorylation in T cells from patients with systemic lupus erythematosus. Deficient expression of the T cell receptor zeta chain. J. Clin. Investig..

[104] Jury E.C., Kabouridis P.S. (2004). T-lymphocyte signalling in systemic lupus erythematosus: A lipid raft perspective. Lupus.

[105] Janes P.W., Ley S.C., Magee A.I. (1999). Aggregation of lipid rafts accompanies signaling via the T cell antigen receptor. J. Cell Biol..

[106] Crispin J.C., Kyttaris V., Juang Y.T., Tsokos G.C. (2007). Systemic lupus erythematosus: New molecular targets. Ann. Rheum. Dis..

[107] Moulton V.R., Tsokos G.C. (2011). Abnormalities of T cell signaling in systemic lupus erythematosus. Arthritis Res. Ther..

[108] McDonald G., Deepak S., Miguel L., Hall C.J., Isenberg D.A., Magee A.I., Butters T., Jury E.C. (2014). Normalizing glycosphingolipids restores function in CD4^+^ T cells from lupus patients. J. Clin. Investig..

[109] Varshney P., Yadav V., Saini N. (2016). Lipid rafts in immune signalling: Current progress and future perspective. Immunology.

[110] Warda M., Tekin S., Gamal M., Khafaga N., Celebi F., Tarantino G. (2025). Lipid rafts: Novel therapeutic targets for metabolic, neurodegenerative, oncological, and cardiovascular diseases. Lipids Health Dis..

[111] Kidani Y., Bensinger S.J. (2014). Lipids rule: Resetting lipid metabolism restores T cell function in systemic lupus erythematosus. J. Clin. Investig..

[112] Zhang X., Lindwall E., Gauthier C., Lyman J., Spencer N., Alarakhia A., Fraser A., Ing S., Chen M., Webb-Detiege T. (2015). Circulating CXCR5+CD4+helper T cells in systemic lupus erythematosus patients share phenotypic properties with germinal center follicular helper T cells and promote antibody production. Lupus.

[113] Craft J.E. (2012). Follicular helper T cells in immunity and systemic autoimmunity. Nat. Rev. Rheumatol..

[114] Simpson N., Gatenby P.A., Wilson A., Malik S., Fulcher D.A., Tangye S.G., Manku H., Vyse T.J., Roncador G., Huttley G.A. (2010). Expansion of circulating T cells resembling follicular helper T cells is a fixed phenotype that identifies a subset of severe systemic lupus erythematosus. Arthritis Rheum..

[115] Linterman M.A., Rigby R.J., Wong R.K., Yu D., Brink R., Cannons J.L., Schwartzberg P.L., Cook M.C., Walters G.D., Vinuesa C.G. (2009). Follicular helper T cells are required for systemic autoimmunity. J. Exp. Med..

[116] Chen P.M., Tsokos G.C. (2021). The role of CD8+ T-cell systemic lupus erythematosus pathogenesis: An update. Curr. Opin. Rheumatol..

[117] Kis-Toth K., Comte D., Karampetsou M.P., Kyttaris V.C., Kannan L., Terhorst C., Tsokos G.C. (2016). Selective Loss of Signaling Lymphocytic Activation Molecule Family Member 4-Positive CD8^+^ T Cells Contributes to the Decreased Cytotoxic Cell Activity in Systemic Lupus Erythematosus. Arthritis Rheumatol..

[118] Jiao Q., Liu C., Yang Z., Ding Q., Wang M., Li M., Zhu T., Qian H., Li W., Tu N. (2014). Upregulated PD-1 Expression Is Associated with the Development of Systemic Lupus Erythematosus, but Not the PD-1.1 Allele of the PDCD1 Gene. Int. J. Genom..

[119] Song L.J., Wang X., Wang X.P., Li D., Ding F., Liu H.X., Yu X., Li X.F., Shu Q. (2015). Increased Tim-3 expression on peripheral T lymphocyte subsets and association with higher disease activity in systemic lupus erythematosus. Diagn. Pathol..

[120] Crispin J.C., Hedrich C.M., Suarez-Fueyo A., Comte D., Tsokos G.C. (2017). SLE-Associated Defects Promote Altered T Cell Function. Crit. Rev. Immunol..

[121] Wherry E.J. (2011). T cell exhaustion. Nat. Immunol..

[122] McKinney E.F., Lee J.C., Jayne D.R., Lyons P.A., Smith K.G. (2015). T-cell exhaustion, co-stimulation and clinical outcome in autoimmunity and infection. Nature.

[123] Yin Y., Choi S.C., Xu Z., Perry D.J., Seay H., Croker B.P., Sobel E.S., Brusko T.M., Morel L. (2015). Normalization of CD4^+^ T cell metabolism reverses lupus. Sci. Transl. Med..

[124] Kyttaris V.C., Zhang Z., Kuchroo V.K., Oukka M., Tsokos G.C. (2010). Cutting edge: IL-23 receptor deficiency prevents the development of lupus nephritis in C57BL/6-lpr/lpr mice. J. Immunol..

[125] Delgoffe G.M., Pollizzi K.N., Waickman A.T., Heikamp E., Meyers D.J., Horton M.R., Xiao B., Worley P.F., Powell J.D. (2011). The kinase mTOR regulates the differentiation of helper T cells through the selective activation of signaling by mTORC1 and mTORC2. Nat. Immunol..

[126] Perl A. (2016). Activation of mTOR (mechanistic target of rapamycin) in rheumatic diseases. Nat. Rev. Rheumatol..

[127] Nagata S. (2018). Apoptosis and Clearance of Apoptotic Cells. Annu. Rev. Immunol..

[128] Platt N., Gordon S. (1998). Scavenger receptors: Diverse activities and promiscuous binding of polyanionic ligands. Chem. Biol..

[129] Platt N., Haworth R., Darley L., Gordon S. (2002). The many roles of the class A macrophage scavenger receptor. Int. Rev. Cytol..

[130] Canton J., Neculai D., Grinstein S. (2013). Scavenger receptors in homeostasis and immunity. Nat. Rev. Immunol..

[131] Peiser L., Mukhopadhyay S., Gordon S. (2002). Scavenger receptors in innate immunity. Curr. Opin. Immunol..

[132] Wermeling F., Chen Y., Pikkarainen T., Scheynius A., Winqvist O., Izui S., Ravetch J.V., Tryggvason K., Karlsson M.C. (2007). Class A scavenger receptors regulate tolerance against apoptotic cells, and autoantibodies against these receptors are predictive of systemic lupus. J. Exp. Med..

[133] Hahn S., Chitre M., Shepard D., Rashid R., Ramirez-Ortiz Z.G. (2025). Scavenger receptors: Key players in the immunological puzzle of lupus. Front. Lupus.

[134] Pluddemann A., Mukhopadhyay S., Gordon S. (2006). The interaction of macrophage receptors with bacterial ligands. Expert. Rev. Mol. Med..

[135] Pluddemann A., Neyen C., Gordon S. (2007). Macrophage scavenger receptors and host-derived ligands. Methods.

[136] Silverstein R.L., Febbraio M. (2009). CD36, a scavenger receptor involved in immunity, metabolism, angiogenesis, and behavior. Sci. Signal..

[137] Greenberg M.E., Sun M., Zhang R., Febbraio M., Silverstein R., Hazen S.L. (2006). Oxidized phosphatidylserine-CD36 interactions play an essential role in macrophage-dependent phagocytosis of apoptotic cells. J. Exp. Med..

[138] Ye Y., Wu T., Zhang T., Han J., Habazi D., Saxena R., Mohan C. (2019). Elevated oxidized lipids, anti-lipid autoantibodies and oxidized lipid immune complexes in active SLE. Clin. Immunol..

[139] Jorge A.M., Lao T., Kim R., Licciardi S., El Khoury J., Luster A.D., Means T.K., Ramirez-Ortiz Z.G. (2022). SCARF1-Induced Efferocytosis Plays an Immunomodulatory Role in Humans, and Autoantibodies Targeting SCARF1 Are Produced in Patients with Systemic Lupus Erythematosus. J. Immunol..

[140] Poon I.K., Lucas C.D., Rossi A.G., Ravichandran K.S. (2014). Apoptotic cell clearance: Basic biology and therapeutic potential. Nat. Rev. Immunol..

[141] Gaipl U.S., Munoz L.E., Grossmayer G., Lauber K., Franz S., Sarter K., Voll R.E., Winkler T., Kuhn A., Kalden J. (2007). Clearance deficiency and systemic lupus erythematosus (SLE). J. Autoimmun..

[142] Herrmann M., Voll R.E., Zoller O.M., Hagenhofer M., Ponner B.B., Kalden J.R. (1998). Impaired phagocytosis of apoptotic cell material by monocyte-derived macrophages from patients with systemic lupus erythematosus. Arthritis Rheum..

[143] Ullal A.J., Reich C.F., Clowse M., Criscione-Schreiber L.G., Tochacek M., Monestier M., Pisetsky D.S. (2011). Microparticles as antigenic targets of antibodies to DNA and nucleosomes in systemic lupus erythematosus. J. Autoimmun..

[144] Elliott M.R., Chekeni F.B., Trampont P.C., Lazarowski E.R., Kadl A., Walk S.F., Park D., Woodson R.I., Ostankovich M., Sharma P. (2009). Nucleotides released by apoptotic cells act as a find-me signal to promote phagocytic clearance. Nature.

[145] Moore K.J., Freeman M.W. (2006). Scavenger receptors in atherosclerosis: Beyond lipid uptake. Arterioscler. Thromb. Vasc. Biol..

[146] Miller Y.I., Chang M.K., Binder C.J., Shaw P.X., Witztum J.L. (2003). Oxidized low density lipoprotein and innate immune receptors. Curr. Opin. Lipidol..

[147] Green D.R., Oguin T.H., Martinez J. (2016). The clearance of dying cells: Table for two. Cell Death Differ..

[148] Psarras A., Clarke A. (2023). A cellular overview of immunometabolism in systemic lupus erythematosus. Oxf. Open Immunol..

[149] Zhang C.X., Wang H.Y., Yin L., Mao Y.Y., Zhou W. (2020). Immunometabolism in the pathogenesis of systemic lupus erythematosus. J. Transl. Autoimmun..

[150] Gergely P., Niland B., Gonchoroff N., Pullmann R., Phillips P.E., Perl A. (2002). Persistent mitochondrial hyperpolarization, increased reactive oxygen intermediate production, and cytoplasmic alkalinization characterize altered IL-10 signaling in patients with systemic lupus erythematosus. J. Immunol..

[151] Tellier J., Shi W., Minnich M., Liao Y., Crawford S., Smyth G.K., Kallies A., Busslinger M., Nutt S.L. (2016). Blimp-1 controls plasma cell function through the regulation of immunoglobulin secretion and the unfolded protein response. Nat. Immunol..

[152] O’Neill L.A., Pearce E.J. (2016). Immunometabolism governs dendritic cell and macrophage function. J. Exp. Med..

[153] Pearce E.L., Pearce E.J. (2013). Metabolic pathways in immune cell activation and quiescence. Immunity.

[154] Oaks Z., Perl A. (2014). Metabolic control of the epigenome in systemic Lupus erythematosus. Autoimmunity.

[155] Lai X., Huang J., Li H., Chang C., Li R., Li X., Yan X., Dong L. (2025). Epigenetic regulatory mechanisms of autoimmune skin diseases: Novel biomarkers and therapeutic prospects. Clin. Epigenet..

[156] Absher D.M., Li X., Waite L.L., Gibson A., Roberts K., Edberg J., Chatham W.W., Kimberly R.P. (2013). Genome-wide DNA methylation analysis of systemic lupus erythematosus reveals persistent hypomethylation of interferon genes and compositional changes to CD4^+^ T-cell populations. PLoS Genet..

[157] Pober J.S., Sessa W.C. (2007). Evolving functions of endothelial cells in inflammation. Nat. Rev. Immunol..

[158] Lui S.L., Yung S., Tsang R., Zhang F., Chan K.W., Tam S., Chan T.M. (2008). Rapamycin prevents the development of nephritis in lupus-prone NZB/W F1 mice. Lupus.

[159] Zhang D., Wang M., Shi G., Pan P., Ji J., Li P. (2020). Regulating T Cell Population Alleviates SLE by Inhibiting mTORC1/C2 in MRL/lpr Mice. Front. Pharmacol..

[160] de Zubiria Salgado A., Herrera-Diaz C. (2012). Lupus nephritis: An overview of recent findings. Autoimmune Dis..

[161] Anders H.J. (2014). Immune system modulation of kidney regeneration--mechanisms and implications. Nat. Rev. Nephrol..

[162] Davidson A., Aranow C. (2006). Pathogenesis and treatment of systemic lupus erythematosus nephritis. Curr. Opin. Rheumatol..

[163] Fu R., Guo C., Wang S., Huang Y., Jin O., Hu H., Chen J., Xu B., Zhou M., Zhao J. (2017). Podocyte Activation of NLRP3 Inflammasomes Contributes to the Development of Proteinuria in Lupus Nephritis. Arthritis Rheumatol..

[164] Wright R.D., Beresford M.W. (2018). Podocytes contribute, and respond, to the inflammatory environment in lupus nephritis. Am. J. Physiol. Ren. Physiol..

[165] Kato H., Perl A. (2014). Mechanistic target of rapamycin complex 1 expands Th17 and IL-4^+^ CD4^ࢤ^CD8^ࢤ^ double-negative T cells and contracts regulatory T cells in systemic lupus erythematosus. J. Immunol..

[166] Crispin J.C., Oukka M., Bayliss G., Cohen R.A., Van Beek C.A., Stillman I.E., Kyttaris V.C., Juang Y.T., Tsokos G.C. (2008). Expanded double negative T cells in patients with systemic lupus erythematosus produce IL-17 and infiltrate the kidneys. J. Immunol..

[167] Wang S., Wang J., Kumar V., Karnell J.L., Naiman B., Gross P.S., Rahman S., Zerrouki K., Hanna R., Morehouse C. (2018). IL-21 drives expansion and plasma cell differentiation of autoreactive CD11c(hi)T-bet(+) B cells in SLE. Nat. Commun..

[168] Roman M.J., Shanker B.A., Davis A., Lockshin M.D., Sammaritano L., Simantov R., Crow M.K., Schwartz J.E., Paget S.A., Devereux R.B. (2003). Prevalence and correlates of accelerated atherosclerosis in systemic lupus erythematosus. N. Engl. J. Med..

[169] Manzi S., Meilahn E.N., Rairie J.E., Conte C.G., Medsger T.A., Jansen-McWilliams L., D’Agostino R.B., Kuller L.H. (1997). Age-specific incidence rates of myocardial infarction and angina in women with systemic lupus erythematosus: Comparison with the Framingham Study. Am. J. Epidemiol..

[170] Esdaile J.M., Abrahamowicz M., Grodzicky T., Li Y., Panaritis C., du Berger R., Cote R., Grover S.A., Fortin P.R., Clarke A.E. (2001). Traditional Framingham risk factors fail to fully account for accelerated atherosclerosis in systemic lupus erythematosus. Arthritis Rheum..

[171] Bertsias G.K., Boumpas D.T. (2010). Pathogenesis, diagnosis and management of neuropsychiatric SLE manifestations. Nat. Rev. Rheumatol..

[172] Jayasinghe M., Rashidi F., Gadelmawla A.F., Pitton Rissardo J., Rashidi M., Elendu C.C., Fornari Caprara A.L., Khalil I., Hmedat K.I., Atef M. (2025). Neurological Manifestations of Systemic Lupus Erythematosus: A Comprehensive Review. Cureus.

[173] Abbott N.J., Mendonca L.L., Dolman D.E. (2003). The blood-brain barrier in systemic lupus erythematosus. Lupus.

[174] Diamond B., Volpe B.T. (2012). A model for lupus brain disease. Immunol. Rev..

[175] Clary D.O., Rothman J.E. (1990). Purification of three related peripheral membrane proteins needed for vesicular transport. J. Biol. Chem..

[176] Kowal C., Diamond B. (2012). Aspects of CNS lupus: Mouse models of anti-NMDA receptor antibody mediated reactivity. Methods Mol. Biol..

[177] Tay S.H., Mak A. (2015). Anti-NR2A/B Antibodies and Other Major Molecular Mechanisms in the Pathogenesis of Cognitive Dysfunction in Systemic Lupus Erythematosus. Int. J. Mol. Sci..

[178] Kivity S., Agmon-Levin N., Zandman-Goddard G., Chapman J., Shoenfeld Y. (2015). Neuropsychiatric lupus: A mosaic of clinical presentations. BMC Med..

[179] Hirohata S., Arinuma Y., Yanagida T., Yoshio T. (2014). Blood-brain barrier damages and intrathecal synthesis of anti-N-methyl-D-aspartate receptor NR2 antibodies in diffuse psychiatric/neuropsychological syndromes in systemic lupus erythematosus. Arthritis Res. Ther..

[180] Stock A.D., Gelb S., Pasternak O., Ben-Zvi A., Putterman C. (2017). The blood brain barrier and neuropsychiatric lupus: New perspectives in light of advances in understanding the neuroimmune interface. Autoimmun. Rev..

[181] Diamond B., Huerta P.T., Mina-Osorio P., Kowal C., Volpe B.T. (2009). Losing your nerves? Maybe it’s the antibodies. Nat. Rev. Immunol..

[182] Cocco C., Manca E., Corda G., Angioni M.M., Noli B., Congia M., Loy F., Isola M., Chessa E., Floris A. (2023). Brain-reactive autoantibodies in neuropsychiatric systemic lupus erythematosus. Front. Immunol..

[183] Nestor J., Arinuma Y., Huerta T.S., Kowal C., Nasiri E., Kello N., Fujieda Y., Bialas A., Hammond T., Sriram U. (2018). Lupus antibodies induce behavioral changes mediated by microglia and blocked by ACE inhibitors. J. Exp. Med..

[184] Kuhn A., Ruland V., Bonsmann G. (2011). Cutaneous lupus erythematosus: Update of therapeutic options part I. J. Am. Acad. Dermatol..

[185] Garelli C.J., Refat M.A., Nanaware P.P., Ramirez-Ortiz Z.G., Rashighi M., Richmond J.M. (2020). Current Insights in Cutaneous Lupus Erythematosus Immunopathogenesis. Front. Immunol..

[186] Hersh A.O., Arkin L.M., Prahalad S. (2016). Immunogenetics of cutaneous lupus erythematosus. Curr. Opin. Pediatr..

[187] Fiehn C. (2017). Familial Chilblain Lupus—What Can We Learn from Type I Interferonopathies?. Curr. Rheumatol. Rep..

[188] Konig N., Fiehn C., Wolf C., Schuster M., Cura Costa E., Tungler V., Alvarez H.A., Chara O., Engel K., Goldbach-Mansky R. (2017). Familial chilblain lupus due to a gain-of-function mutation in STING. Ann. Rheum. Dis..

[189] Rice G., Newman W.G., Dean J., Patrick T., Parmar R., Flintoff K., Robins P., Harvey S., Hollis T., O’Hara A. (2007). Heterozygous mutations in TREX1 cause familial chilblain lupus and dominant Aicardi-Goutieres syndrome. Am. J. Hum. Genet..

[190] Zimmermann N., Wolf C., Schwenke R., Luth A., Schmidt F., Engel K., Lee-Kirsch M.A., Gunther C. (2019). Assessment of Clinical Response to Janus Kinase Inhibition in Patients With Familial Chilblain Lupus and TREX1 Mutation. JAMA Dermatol..

[191] Gunther C., Berndt N., Wolf C., Lee-Kirsch M.A. (2015). Familial chilblain lupus due to a novel mutation in the exonuclease III domain of 3′ repair exonuclease 1 (TREX1). JAMA Dermatol..

[192] Okon L.G., Werth V.P. (2013). Cutaneous lupus erythematosus: Diagnosis and treatment. Best Pract. Res. Clin. Rheumatol..

[193] Kuhn A., Sticherling M., Bonsmann G. (2007). Clinical manifestations of cutaneous lupus erythematosus. J. Dtsch. Dermatol. Ges..

[194] Ewald S.E., Barton G.M. (2011). Nucleic acid sensing Toll-like receptors in autoimmunity. Curr. Opin. Immunol..

[195] Gunther C. (2019). Nucleic Acid Immunity in the Pathogenesis of Cutaneous Lupus Erythematosus. Front. Immunol..

[196] Wang Q., Chen M., Jin H., Xiao J., Zhang Y. (2026). Persistent cutaneous lupus erythematosus: A pathway toward systemic disease?. J. Transl. Autoimmun..

[197] Wenzel J. (2019). Cutaneous lupus erythematosus: New insights into pathogenesis and therapeutic strategies. Nat. Rev. Rheumatol..

[198] Neff H.A., Yildiz-Altay U., Salam N., Ward D.V., Shepard D., Ramirez-Ortiz Z.G., Richmond J.M. (2026). Gut dysbiosis in a murine model of cutaneous lupus erythematosus correlates with antigen-specific T cells and antigen-presenting cells in skin. Sci. Rep..

[199] Hevia A., Milani C., Lopez P., Cuervo A., Arboleya S., Duranti S., Turroni F., Gonzalez S., Suarez A., Gueimonde M. (2014). Intestinal dysbiosis associated with systemic lupus erythematosus. mBio.

[200] Shepard D.M., Hahn S., Chitre M., Neff H., Ward D.V., Jadhav N., Richmond J.M., Ramirez-Ortiz Z.G. (2026). SCARF1 deficiency exacerbates gut inflammation and autoimmune pathology. Sci. Rep..

[201] Belkaid Y., Hand T.W. (2014). Role of the microbiota in immunity and inflammation. Cell.

[202] Honda K., Littman D.R. (2016). The microbiota in adaptive immune homeostasis and disease. Nature.

[203] Mu C., Yang Y., Zhu W. (2015). Crosstalk Between The Immune Receptors and Gut Microbiota. Curr. Protein Pept. Sci..

[204] Manfredo Vieira S., Hiltensperger M., Kumar V., Zegarra-Ruiz D., Dehner C., Khan N., Costa F.R.C., Tiniakou E., Greiling T., Ruff W. (2018). Translocation of a gut pathobiont drives autoimmunity in mice and humans. Science.

[205] Zegarra-Ruiz D.F., El Beidaq A., Iniguez A.J., Lubrano Di Ricco M., Manfredo Vieira S., Ruff W.E., Mubiru D., Fine R.L., Sterpka J., Greiling T.M. (2019). A Diet-Sensitive Commensal Lactobacillus Strain Mediates TLR7-Dependent Systemic Autoimmunity. Cell Host Microbe.

[206] Baechler E.C., Batliwalla F.M., Karypis G., Gaffney P.M., Ortmann W.A., Espe K.J., Shark K.B., Grande W.J., Hughes K.M., Kapur V. (2003). Interferon-inducible gene expression signature in peripheral blood cells of patients with severe lupus. Proc. Natl. Acad. Sci. USA.

[207] Kirou K.A., Lee C., George S., Louca K., Peterson M.G., Crow M.K. (2005). Activation of the interferon-alpha pathway identifies a subgroup of systemic lupus erythematosus patients with distinct serologic features and active disease. Arthritis Rheum..

[208] Khamashta M., Merrill J.T., Werth V.P., Furie R., Kalunian K., Illei G.G., Drappa J., Wang L., Greth W. (2016). Sifalimumab, an anti-interferon-alpha monoclonal antibody, in moderate to severe systemic lupus erythematosus: A randomised, double-blind, placebo-controlled study. Ann. Rheum. Dis..

[209] Morand E.F., Furie R., Tanaka Y., Bruce I.N., Askanase A.D., Richez C., Bae S.C., Brohawn P.Z., Pineda L., Berglind A. (2020). Trial of Anifrolumab in Active Systemic Lupus Erythematosus. N. Engl. J. Med..

[210] Christensen S.R., Shupe J., Nickerson K., Kashgarian M., Flavell R.A., Shlomchik M.J. (2006). Toll-like receptor 7 and TLR9 dictate autoantibody specificity and have opposing inflammatory and regulatory roles in a murine model of lupus. Immunity.

[211] Merrill J.T., Neuwelt C.M., Wallace D.J., Shanahan J.C., Latinis K.M., Oates J.C., Utset T.O., Gordon C., Isenberg D.A., Hsieh H.J. (2010). Efficacy and safety of rituximab in moderately-to-severely active systemic lupus erythematosus: The randomized, double-blind, phase II/III systemic lupus erythematosus evaluation of rituximab trial. Arthritis Rheum..

[212] Mackay F., Schneider P., Rennert P., Browning J. (2003). BAFF AND APRIL: A tutorial on B cell survival. Annu. Rev. Immunol..

[213] Wofsy D., Hillson J.L., Diamond B. (2012). Abatacept for lupus nephritis: Alternative definitions of complete response support conflicting conclusions. Arthritis Rheum..

[214] He J., Zhang X., Wei Y., Sun X., Chen Y., Deng J., Jin Y., Gan Y., Hu X., Jia R. (2016). Low-dose interleukin-2 treatment selectively modulates CD4(+) T cell subsets in patients with systemic lupus erythematosus. Nat. Med..

[215] Fernandez D., Bonilla E., Mirza N., Niland B., Perl A. (2006). Rapamycin reduces disease activity and normalizes T cell activation-induced calcium fluxing in patients with systemic lupus erythematosus. Arthritis Rheum..

